# Adipose methylome integrative-omic analyses reveal genetic and dietary metabolic health drivers and insulin resistance classifiers

**DOI:** 10.1186/s13073-022-01077-z

**Published:** 2022-07-18

**Authors:** Colette Christiansen, Max Tomlinson, Melissa Eliot, Emma Nilsson, Ricardo Costeira, Yujing Xia, Sergio Villicaña, Olatz Mompeo, Philippa Wells, Juan Castillo-Fernandez, Louis Potier, Marie-Claude Vohl, Andre Tchernof, Julia El-Sayed Moustafa, Cristina Menni, Claire J. Steves, Karl Kelsey, Charlotte Ling, Elin Grundberg, Kerrin S. Small, Jordana T. Bell

**Affiliations:** 1 Department of Twin Research and Genetic Epidemiology, King’s College London, London, UK; 2 Department of Medical & Molecular Genetics, King’s College London, London, UK; 3 Department of Epidemiology, Brown University School of Public Health, Providence, R.I. USA; 4 Epigenetics and Diabetes Unit, Department of Clinical Sciences, Lund University Diabetes Centre, Lund University, Scania University Hospital, Malmö, Sweden; 5 Diabetology Department, Bichat Hospital, AP-HP, Université de Paris, Paris, France; 6 Institute of Nutrition and Functional Foods (INAF), Université Laval, Québec, QC Canada; 7 Québec Heart and Lung Institute, Université Laval, Québec, QC Canada; 8 Genomic Medicine Center, Children’s Mercy Research Institute, Children’s Mercy Kansas City, Kansas City, MO 64108 USA

**Keywords:** DNA methylation, Visceral fat, Adiposity, Integrative omics

## Abstract

**Background:**

There is considerable evidence for the importance of the DNA methylome in metabolic health, for example, a robust methylation signature has been associated with body mass index (BMI). However, visceral fat (VF) mass accumulation is a greater risk factor for metabolic disease than BMI alone. In this study, we dissect the subcutaneous adipose tissue (SAT) methylome signature relevant to metabolic health by focusing on VF as the major risk factor of metabolic disease. We integrate results with genetic, blood methylation, SAT gene expression, blood metabolomic, dietary intake and metabolic phenotype data to assess and quantify genetic and environmental drivers of the identified signals, as well as their potential functional roles.

**Methods:**

Epigenome-wide association analyses were carried out to determine visceral fat mass-associated differentially methylated positions (VF-DMPs) in SAT samples from 538 TwinsUK participants. Validation and replication were performed in 333 individuals from 3 independent cohorts. To assess functional impacts of the VF-DMPs, the association between VF and gene expression was determined at the genes annotated to the VF-DMPs and an association analysis was carried out to determine whether methylation at the VF-DMPs is associated with gene expression. Further epigenetic analyses were carried out to compare methylation levels at the VF-DMPs as the response variables and a range of different metabolic health phenotypes including android:gynoid fat ratio (AGR), lipids, blood metabolomic profiles, insulin resistance, T2D and dietary intake variables. The results from all analyses were integrated to identify signals that exhibit altered SAT function and have strong relevance to metabolic health.

**Results:**

We identified 1181 CpG positions in 788 genes to be differentially methylated with VF (VF-DMPs) with significant enrichment in the insulin signalling pathway. Follow-up cross-omic analysis of VF-DMPs integrating genetics, gene expression, metabolomics, diet, and metabolic traits highlighted VF-DMPs located in 9 genes with strong relevance to metabolic disease mechanisms, with replication of signals in *FASN*, *SREBF1*, *TAGLN2*, *PC* and *CFAP410*. *PC* methylation showed evidence for mediating effects of diet on VF. *FASN* DNA methylation exhibited putative causal effects on VF that were also strongly associated with insulin resistance and methylation levels in *FASN* better classified insulin resistance (AUC=0.91) than BMI or VF alone.

**Conclusions:**

Our findings help characterise the adiposity-associated methylation signature of SAT, with insights for metabolic disease risk.

**Supplementary Information:**

The online version contains supplementary material available at 10.1186/s13073-022-01077-z.

## Background

Obesity is a global health concern, where the number of obese adults worldwide has tripled since 1975 to 13% in 2016 [1] and is predicted to continue to rise [2]. Obesity has a pronounced impact on an individual’s health, with obese individuals more likely to suffer from type 2 diabetes (T2D), heart disease and cancer [3, 4], resulting in a significant economic impact [5].

Despite evidence for a clear genetic component to obesity, with GWAS studies identifying over 200 genetic variants, these explain only 2–3% of variability in obesity between individuals [6]. The dramatic rise in obesity in recent years suggests involvement of environmental drivers. Epigenetic mechanisms are key regulators of gene function that can respond to external stimuli. Multiple epigenome-wide association studies of obesity have been carried out, with the majority of studies in blood [7] and relatively few investigating the adipose tissue methylome, which is one of the most biologically relevant tissues for metabolic health.

Several SAT methylome studies of obesity have been carried out to date [8–11], each in up to 250 participants across different ancestries and with different methylation technologies. A clear and strong adipose methylome signature of obesity has emerged, with subsets of signals also relating to glucose and insulin homeostasis, lipid metabolism, and type 2 diabetes (T2D). Ronn et al. [9] identified thousands of strong associations between BMI and the adipose tissue methylome in approximately 200 Northern Europeans, but without adjustment for adipose tissue cell heterogeneity. In contrast, Agha et al. [8] found that after adjustment for adipose cell composition there were no significant associations with BMI, but associations were observed with other aspects of adiposity such as android:gynoid fat ratio (AGR) in 100 participants of mixed ethnicity. Orozco et al. [10] identified adipose methylation signals associated with one or more of 32 traits related to obesity and T2D in 200 Finnish men and based on these results developed a T2D classifier. Most recently, Sharma et al. [11] detected over a hundred adipose methylation signals related to BMI and measures of insulin sensitivity in 230 African Americans. Furthermore, a clear genetic influence on the adipose methylome has also been identified [12, 13], and GWAS signals for BMI exhibit strong impacts on adipose tissue methylome variation [12]. These observations suggest that adipose tissue DNA methylation levels not only show major alterations with obesity but may also in part mediate some genetic risk effects in obesity.

However, the majority of adipose tissue methylome studies of obesity to date focus on BMI, which can be an imprecise measure of adiposity without distinction between lean and fat mass. In contrast, estimates of visceral fat (VF) mass accumulation around the abdominal organs have been shown to confer stronger risk for metabolic disease than BMI alone [14]. For example, VF was associated with impaired fasting glucose [15], hypertension and metabolic syndrome [16], while general adiposity was not. Multiple studies have linked VF to impaired insulin signalling and insulin resistance (IR) [17–19]. VF is correlated with liver fat accumulation and is prone to inflammation [17], both of which lead to reduced insulin signalling functionality, although the precise underlying molecular mechanisms are not fully characterised. Furthermore, methylome profiling of visceral adipose tissue in 199 severely obese individuals has revealed hundreds of differentially methylated signals related to circulating lipid levels, with both tissue-specific and shared effects [20].

Here we aimed to dissect subcutaneous adipose tissue methylome alterations relevant to metabolic health, by focusing on visceral fat accumulation as the major risk factor for metabolic disease. We characterised the SAT DNA methylation signature associated with VF after controlling for BMI in 538 twins from the TwinsUK cohort [21]. At the majority of identified signals, DNA methylation levels were concordant across SAT and visceral adipose tissue (VAT), with consistent levels of gene expression across these two types of adipose tissue. We then conducted a multi-omic follow-up analysis integrating our signals with genetic, blood epigenetic, SAT gene expression, diet, plasma metabolomic variation, and a range of metabolic phenotypes to assess and quantify genetic and environmental drivers of the identified signals, as well as their potential functional roles. Replication of components of the analysis was pursued in three independent datasets (104 individuals from the LEAP cohort in the New England Family Study [8], 28 T2D cases and 28 controls from the Danish Twin Registry [22], and 199 severely obese individuals undergoing bariatric surgery at the Quebec Heart and Lung institute [20]). To consider how our results relate to the clinical consequences of elevated VF, we showed that a subset of the identified signals have strong associations with insulin resistance and can serve as potential predictors of IR.

## Methods

### Cohort sample datasets

The primary dataset in this study included 538 White British female twins (mean age 58.9±9.5 years; Table 1) from the TwinsUK cohort [23] who were free from major diseases including cancer, and for whom SAT biopsy DNA methylation levels were profiled. Three subsets of the 538 primary TwinsUK dataset were included in downstream follow-up analyses where participants had complete relevant data (including 397 of 538 for diet analyses, 347 of 538 for metabolomic analyses, and 528 of 538 for lipid analyses). Details of data collection for the diet, metabolomic and lipid data have been previously described [23] and are described further below (see TwinsUK phenotype data). A second set of TwinsUK participants included 901 individuals (mean age 57.8±10.1 years 97% female; Table 1) for whom whole blood DNA methylation was profiled. These data are described further below (see TwinsUK study participants). The final TwinsUK dataset included in this study contained 720 participants from the TwinsUK cohort for whom adipose gene expression profiles were available, and these data were used in the analysis of the association between gene expression and visceral fat described previously [24], and below (see Gene expression profiles and analyses).

**Table 1:** Discovery sample participant characteristics

Tissue type	Blood	SAT
**Number**	901	538
**Smoker status**	109(S)	54(S)
	283(Ex)	196(Ex)
	509(N)	288(N)
**% Female**	97%	100%
**Age (years) ± SD**	57.8±10.1	58.9±9.5
**BMI (kg/m**^**2**^**)**	26.7±4.9	26.8 ±4.9
**Visceral fat (g)**	3117±1383	3198±1441
**Correlation between BMI and visceral fat**	0.87	0.90
**AGR**	n/a	0.96±0.16
**VF/TPM**	n/a	0.109±0.022

Validation and replication were carried out in three independent cohort datasets that are described in more detail below (see ‘Validation and replication’). For validation using AGR as a surrogate adiposity phenotype, we analysed a dataset of 104 individuals from the LEAP cohort in the New England Family Study, as previously described [8]. For replication of metabolic health impacts, two further datasets were used. These included 28 T2D cases and 28 controls from the Danish Twin Registry as previously described [22], and 199 severely obese individuals undergoing bariatric surgery at the Quebec Heart and Lung institute as previously described [20].

A complete analysis plan including datasets, sample sizes and analysis carried out on each dataset can be found in Additional file 1: Fig. S1.

### TwinsUK study participants

The primary dataset of 538 participants consisted of 107 monozygotic (MZ) twin pairs and 163 dizygotic (DZ) twin pairs from whom SAT biopsy samples were obtained, as previously described [21]. Briefly, subcutaneous tissue was dissected from the abdomen, near the umbilicus. Fat was immediately stored in liquid nitrogen, after separation from the skin layer. The second set of 901 study participants consisted of 422 MZ twin pairs and 73 DZ twin pairs, and 222 individuals overlapped with the set of 538 twins with SAT biopsies. Descriptions of DNA extraction in blood samples and SAT have been described previously [21, 25]. All study participants provided informed consent. Ethical approval was granted by the National Research Ethics Service London-Westminster, the St Thomas’ Hospital Research Ethics Committee.

### TwinsUK phenotype data

The individuals included in the study attended clinical research visits during which phenotype data and biological samples were collected. During the visit height and weight were measured, and dual-energy X-ray absorptiometry whole-body scans (DXA) were carried out. The total grams of VF obtained from the trunk region in the DXA data were used to quantify VF, as previously described [26, 27]. Briefly, DXA scans were undertaken with participants lying flat and straight, and the relevant fat mass was estimated in grams from a cross section of the whole body at L4–L5, the typical location of a CT slice. The procedure for taking these measurements, quality control and the calculation of VF has previously been described [26, 27]. Only individuals with complete information for height, weight and VF were used in the analysis. DXA scans were also used to estimate AGR and trunk fat mass (TFM).

Fasting insulin and glucose levels were obtained from all 538 participants’ whole blood samples collected during the clinical visits. Insulin resistance (IR) and type 2 diabetes (T2D) status were assessed through both questionnaire data and based on blood glucose and insulin levels. For an individual to be classified as insulin resistant, they had at least two normal fasting blood glucose readings (below 7 mmol/L) accompanied by a fasting insulin level > 50 pmol/L or over 9 μU/ml. Individuals with only one instance meeting these thresholds were removed from the controls. This categorisation resulted in 114 IR cases (HOMA-IR 3.5 ± 1.5) and 284 IR controls (HOMA-IR 1.1 ± 0.5) in the SAT dataset.

An individual was categorised as a T2D case if either there were two or more indicators of T2D or T2D medication was listed in regular medications taken. Indicators included fasting blood glucose levels greater than 7 mmol/L on any occasion and self-reporting of T2D incidence. Altogether, the SAT dataset included 15 T2D cases and 378 T2D controls.

Lipid data were available for 528 twins with SAT methylation profiles. Total cholesterol, HDL, LDL and triglycerides were measured in blood serum in mmol/L. Total cholesterol, HDL and triglycerides were determined by a colorimetric enzymatic method. HDL cholesterol was determined after precipitation of larger particles (chylomicron, VLDL and LDL) by magnesium and dextran sulfate. LDL levels were estimated by using the Friedewald equation. Checks were carried out to ensure the date difference between the date of methylation measurement and lipid collection was within 5 years. Outliers were excluded based on a boxplot of lipid measurements, where data points that are located outside the whiskers of the boxplot are excluded. This left 525 subjects in the downstream analysis for HDL, 499 for triglycerides, 527 for total cholesterol and 521 for LDL.

Fasting serum circulating metabolomic profiles were measured by Nightingale Health Ltd, formerly known as Brainshake Ltd (Vantaa, Finland; https://nightingalehealth.com/services) [28]. Metabolomic profiles were determined using targeted NMR spectroscopy, as previously described [28]. Metabolomic profiles were obtained for 12 metabolic traits, 143 metabolite concentrations, 80 lipid ratios, 3 lipoprotein sizes and a measure of albumin. Metabolomic data processing in the larger TwinsUK cohort sample has been previously described [29], and briefly traits were log-transformed and scaled to standard deviation units. This component of the analysis focused on 347 of the 538 twins, including only individuals who had metabolomic profiles within 5 years of the SAT biopsy.

### SAT and blood cell composition estimates

SAT cell composition proportions were estimated based on previously developed approaches using gene expression profiles with CIBERSORT [24]. The estimated proportion of adipocytes, macrophages and micro-vascular endothelial cells were included as covariates in all downstream analyses involving SAT. Blood cell subtype proportions were estimated based on DNA methylation profiles, using established methods [30]. Blood cell estimates were obtained for monocytes, granulocytes, immune cells (Natural Killer (NK) cells, CD8 and CD4) and plasmablasts. As the resulting cell subtype proportions were correlated, downstream analysis models included only monocytes, granulocytes, NK cells and CD8 naïve cells.

### TwinsUK DNA methylation profiles and analyses

DNA methylation profiles for both SAT and whole blood in twins were generated using the Ilumina HumanMethylation 450kBeadChip array (450k array) [31]. DNA methylation levels were determined using methylation beta-values, defined as the ratio of the methylated bead signal to the sum of the unmethylated bead signal plus the methylated bead signal plus 100 [32]. Methylation beta-values range between 0 at unmethylated CpG sites and 1 at fully methylated CpG sites. Enmix [33] was used for methylation data processing and quality control. ENmix was first applied for background and dye bias correction quantile normalisation of signals, and estimation of adjusted beta-values. Missing data was assigned for CpG signals with detP > 0.000001 and Nbead < 3. Sample outliers were excluded as missing data using standard parameters. Minfi [34] was used to exclude samples with median methylated and unmethylated signals below 10.5. Cross-reactive probes and probes containing and >2 alignment mismatches were excluded. Altogether, 438,594 probes were included in the downstream analysis.

Epigenome-wide association scans (EWAS) were carried out for VF in SAT both with and without BMI as a covariate. DNA methylation values for each CpG site were normalised to N(0,1) prior to fitting linear models. Mixed effect linear models were fitted (using lme4 and LmerTest in R) [35] where methylation was the response variable, and VF was the predictor and fixed effect covariates were BMI, age, smoking status, cell type proportion and methylation chip and position of the sample on the chip, random effects covariates were zygosity, family. Multiple testing adjustment was performed using a Bonferroni adjusted threshold of 5% (*P* = 1.14 × 10^−7^). Methylation effect sizes were calculated using the same linear model, but without normalising DNA methylation levels to N(0,1) prior to data analysis, and the results were also compared to the normalised analysis. The association between VF and DNA methylation levels was also assessed over larger genomic regions, to identify VF differentially methylated region (VF-DMR) using DMRcate [36, 37]. VF-DMRs were identified after correction for multiple testing genome-wide, based on DMRcate Fisher FDR 5%.

Epigenetic analyses were also carried out to relate methylation levels at the 1181 VF-DMPs as the response variables and a range of different metabolic health phenotypes including AGR, VF/TFM, lipids, blood metabolomic profiles, insulin resistance and T2D. Overlap between the VF-DMPs and methylation association signals from each metabolic phenotype were established using a *P*-value cut off based on a Bonferroni multiple testing threshold (*P* = 4.2 × 10^−5^).

SAT epigenetic age analyses applied three different epigenetic ageing calculators to estimate DNA methylation age acceleration for each individual, based on DNAm GrimAge [38], DNAm PhenoAge [39] and Horvath methylation age [40]. Mixed effect linear models were fitted (using lme4 and LmerTest in R), where the DNA methylation age acceleration was the response variable with predictor visceral fat. Fixed effect covariates included age, smoking status and cell type composition and mixed effects included zygosity and family. The analysis was run with and without BMI as a fixed effect covariate.

### Genome annotation and pathway analysis

CpG annotation to genes and with respect to CpG density was based on the 450k Illumina manifest. Further genomic annotations were carried out taking into account data from the ENCODE project [41] and ChromHMM categorisation [42]. This enabled an assessment of the enrichment or depletion of the differentially methylated signals relative to all tested methylation probes from the 450k array. The number of VF-DMPs mapping to each annotation category (e.g. insulators) was compared to the total number of CpGs tested mapping to that category. Fisher’s exact test was used to determine whether differences were significant, with a *P*-value threshold of *P* < 0.05.

To explore functional annotations to biological processes and molecular pathways, the genes that VF-DMPs annotated to were analysed with the Ingenuity Pathway Analysis (QIAGEN Inc. https://www.qiagenbioinformatics.com/products/ingenuitypathway-analysis) and GOmeth in missMethyl [43]. Using IPA, we assessed evidence for enrichment of canonical pathways for the VF-DMP genes against all genes applying Fisher’s exact test. We considered IPA pathway results that surpassed a *P*-value threshold (*P* < 0.01). We also applied the GOmeth function in the missMethyl Bioconductor package in R [43]. In GOmeth, we tested the 1181 significant CpGs against a background of all 438,594 CpGs included in the downstream analysis for enrichment in KEGG pathways. GOmeth includes allowance in the enrichment calculation for the number of probes per gene in the background set.

### Tissue specificity

The SAT samples included in the study consisted of subcutaneous fat. Previously published datasets [44, 45] were used to explore differences between subcutaneous fat methylation and visceral fat methylation levels at the VF-DMPs. Our first assessment [44] estimated the number of VF-DMPs that fell in genomic regions that showed over 10% methylation differences between the SAT and VAT [44]. Our second assessment explored how many VF-DMPs were also previously identified by Macartney-Coxon et al. [45] to be differentially methylated after multiple testing adjustment between SAT and VAT.

To assess the level of tissue specificity across SAT and blood samples, we analysed the VF-methylation associations at the 1181 VF-DMPs in the 901 whole blood twin samples, with and without taking into account BMI. The resulting *P*-values were used to estimate their *π*0, the overall proportion of true null hypotheses in all tests performed, using Bioconductor qvalue [46]. We then quantified the proportion of significant results *π*1, or the proportion of true positives, corresponding to *π*1 = 1 − *π*0 [47]. For the 222 individuals for whom both blood and SAT methylation data were available, pairwise adipose-blood methylation correlations were estimated at the VF-DMPs to further assess the level of tissue specificity.

### Gene expression profiles and analyses

RNA-seq data [48] were available for 720 individuals with available genotype information (median age 60, age range 38–64, median BMI 25, BMI range 16–47), and these included the 538 twins in the current study. RNA-seq generation and pre-processing in the SAT samples have been previously described [24]. In summary, STAR software v2.4.0.1 [49] was used to align reads to hg19. Samples were excluded if they had less than 10 million reads sequenced to known genes. Samples were also excluded if reads were not properly paired. Gene counts were transformed into trimmed mean of M-values (TMM)-adjusted counts per million (CPMs) and inverse-normalised prior to all downstream analyses. The expression dataset was filtered to a minimum of 5 gene counts in 25% of the subjects.

Two analyses were carried out to assess the functional impacts of the 1181 VF-DMPs. Firstly, the association between VF and gene expression was determined at the 788 genes using a mixed effect linear model. The model applied gene expression level as the response variable and VF as the predictor, with fixed effect covariates smoking, BMI, age, insert size and GC content and random effects covariates primer index, date of sequencing sample, processing batch, family and zygosity. Secondly, an association analysis was carried out to determine whether methylation at the VF-DMPs is associated with gene expression. A mixed effect linear model for the 538 individuals was constructed with gene expression as the response variable and methylation as the predictor. Fixed and random effect covariates were consistent with the analysis above, but also included VF as a fixed effect covariate (in addition to fixed effect covariates smoking, BMI, age, insert size and GC content and random effects covariates, primer index, date of sequencing sample processing batch, family and zygosity). The strength of association was determined using LmerTest [35], consistent with the EWAS analysis.

To explore whether gene expression or methylation was the likely driver of the VF associations, the association model between VF and gene expression above was rerun for the subset of 538 individuals with both methylation and expression data available. Methylation was then added to the model as a covariate and the significance (*P*-value) of the association between gene expression and VF was determined. The extent to which the *P*-value attenuated was observed. This analysis was also repeated starting with the significance of the association between methylation and VF, adding gene expression to the model and observing the extent to which the *P*-value attenuated.

### Genetic data, heritability and QTL analyses

A twin-based heritability analysis was carried out to estimate heritability of the 1181 VF-DMPs using ACE modelling [50], which assumes that phenotypic variance is the sum of additive genetic effects (A), common environmental effects (C) and unique environmental effects (E). Genotypes were available for all 538 individuals in the sample and were used for the identification of methylation Quantitative Trait Loci (meQTLs) and expression Quantitative Trait Loci (eQTLs). Genotyping of the full TwinsUK genetic dataset has been described previously [51]. Briefly HumanHap300, HumanHap610Q, HumanHap1M Duo and HumanHap1.2M Duo 1M arrays were used to genotype the sample. Following pre-phasing using IMPUTE2 without a reference panel, the resulting haplotypes were used to perform fast imputation from 1000 Genome phase1 dataset. Following imputation, quality control measures included the exclusion of SNPs which failed Hardy Weinberg equilibrium (*P* < 1e−6), had a MAF < 0.01, had missingness of more than 5% or had an info score < 0.8. Individuals with discordant sex were removed. Outliers were also removed using PLINK 2.0 (unrelated participants) and GENESIS (related participants) where a deviation of more than 7 SD from the mean was considered an outlier. The data was further pruned for relatedness with participants with IBS > 0.125 (calculated using PLINK 2.0) removed.

A meQTL analysis was performed to test for the association between genetic variation and DNA methylation levels at the 1181 VF-DMPs in SAT. SNPs that were meQTLs were determined by fitting a linear model in MatrixEQTL R package [52], where the corrected methylation beta-values were the response variables, and dosage of minor allele was the predictor. Covariates included age, predicted smoking status, genetic principal components as fixed effects and family relatedness as a random effect. Only *cis* meQTL SNPs were included, where the *cis* interval was defined as ± 1 Mb from CpG site. A stringent *cis* meQTL *P*-value threshold was used to test for significance (*P* = 1 × 10^−5^), as previously described [12], and the most associated SNP per CpG site was reported as the meQTL for the VF-DMP.

We assessed the overlap between the VF-DMP meQTLs SNPs and previously identified GWAS signals for VF from the GWAS catalog [53]. Altogether, all 71 recorded GWAS studies in the catalogue for VF (26) or BMI-adjusted waist-to-hip ratio (45) were included. The studies identified 360 and 3172 SNPs associated with VF and BMI-adjusted waist-to-hip ratio respectively. VF-DMP meQTLs SNPs were compared against this GWAS combined set of 3532 SNPs to explore overlaps.

We further analysed the association between genotype at the most significant SNP for each of the 203 meQTLs and VF in our sample of 538 individuals. A mixed effect linear model (lme4) was fitted for all 538 participants with VF as the response variable, dosage of the minor allele as predictor, and covariates included BMI, age, smoking status as fixed effects and family and zygosity as random effects. Due to the small sample size, 8 nominally significant SNPs (*P* < 0.05) were used in the downstream Mendelian randomisation analysis.

A ratio estimator approach was used for Mendelian randomisation [54]. Here, the causal estimate was calculated as the effect size of the association between genotype and VF, divided by the effect size of the association between genotype and methylation. The standard error of the estimate was calculated using the delta method based on Taylor expansion. Finally using the standard error, a 95% confidence interval was constructed around the causal estimate. Where this interval did not include zero, the causal estimate was deemed to be significant. The assumptions for Mendelian randomisation were assessed, and the assumption that the SNP (meQTL) is associated with the risk factor (VF) is met by design. However, the second and third assumptions are less clearly met, as there could be additional unknown links between genotype and VF.

In addition to the meQTL analysis, a *cis* eQTL analysis was also carried out for the 788 genes annotated to the VF-DMPs in the larger sample of 720 individuals from the TwinsUK cohort with available gene expression data. Genetic effects at SNPs for each gene in a *cis*-window (a 1-MB region around the transcription start site (TSS) of each gene) were analysed. SNPs with a MAF <5% were excluded. As described in Glastonbury et al. [24], the analysis was performed on the inverse-rank-normalised gene expression residuals corrected for family and zygosity and RNA extraction batch. The QTLtools package in R [55] was used for the analysis, adjusting for BMI, genotyping chip and 40 PEER factors. For consistency with meQTL results, *cis* eQTL significant threshold matched that applied for *cis* meQTLs (*P*-value = 1 × 10^−5^). To assess co-localisation of meQTLs and eQTLs, a simplified approach was taken, where a direct comparison was made between meQTL SNPs and eQTL SNPs.

### Diet data and analyses

Dietary information was available for 397 of the 538 participants with SAT methylation. The data were based on the 131 item EPIC-Norfolk food frequency questionnaire (FFQ [56]) where FFQ data processing in the larger TwinsUK sample has previously described [57]. Briefly, the 131 food items were combined to form 54 food groups, with intake for each group estimated as the sum of all weekly servings. Data quality control was performed based on a comparison between the energy intake implied by the FFQ and the participants’ estimated basal metabolic rate. We analysed both nutrient intakes and overall diet quality estimated by diet scores. Nutrient intake variables explored here included biotin, cholesterol, magnesium, fibre, protein, total sugars, total trans fat, tryptophan and vitamin E intakes, all of which were previously identified to be associated with VF [26]. Overall diet quality measures included the Healthy Eating Index (HEI) [58], the alternate Healthy Eating Index (aHEI) [59], the Dietary Approaches to Stop Hypertension (DASH) score [60], the Alternate Mediterranean Diet Score (aMED) [61], the Dietary Quality Index International (DQI-I) [62] ], Health Eating Index (HDI) score [63] and the Nordic Diet Score [64].

Diet epigenetic analyses were carried out assessing the association between methylation levels at the 1181 VF-DPs as the response variables and selected dietary intake variables including nutrient intakes previously associated with VF (biotin, cholesterol, magnesium, fibre, protein, sugars, trans fats, tryptophan, vitamin E; based on Le Roy et al. [26]), and estimated diet scores (HEI, aHEI, DASH, aMED, DQI-I, HDI, Nordic) as predictors. A consistent linear model was used to assess the methylation-diet associations with covariates including age, BMI, smoking status, cell composition, batch effects as fixed effects, family and zygosity as random effects. To assess evidence for significant VF-DMP associations with dietary variables, we considered results that surpassed Bonferroni multiple testing correction (*P* = 4.2 × 10^−5^).

To assess the mediation effect of methylation on the impact of diet on VF, we carried out a mediation analysis using the mediation package in R [65]. Methylation at CpGs identified to harbour differential methylation effects for both VF and diet (VF/diet-DMPs) was used as a mediator for the causal effect of the relevant diet variable on VF. Before undertaking a mediation analysis, the association between VF and the dietary variables where overlapping methylation associations were found (fibre, magnesium, DASH score, Nordic score, DQI-I score) was determined. A consistent linear mixed effect model (lme4) was used with VF as response variable, the dietary variable as predictor, and fixed effect covariates including age, smoking, BMI, age, cell composition and batch, and random effects covariates zygosity and family. All of the dietary variables were significantly associated with VF at *P* <0.05 (Additional file 2: Table S5).

The R mediation package uses a single mediator and fibre, magnesium and DASH diet score all had more than one associated VF/diet-DMP. For each of these dietary variables, the CpG site with the most significant association with the diet variable was used as the mediator. Outcome models were constructed using mixed effects models (lme4) with the diet variable as the predictor and fixed effect covariates smoking, age, BMI, cell composition, batch and random effect covariates family and zygosity. Mediator models were constructed using the same approach with an additional predictor including DNA methylation levels at the relevant CpG site. For each mediation analysis, we report the average causal mediation effect (ACME), representing the average size of the effect of a diet variable on VF that is mediated by methylation. We also report the proportion of the effect that is mediated. We then report the average direct effect (ADE), where ADE represent the direct effect of the diet variable on VF. If only ACME is significant, there is a full mediation by methylation of the association between the diet variable and VF. If both ADE and ACME are significant, methylation has a partial mediating effect.

### Insulin resistance classifier

We tested whether insulin resistance status could be predicted using DNA methylation levels at the most significantly differentially methylated site associated with IR, namely in *FASN* (cg11950105). The analyses were carried out in the IR data set of 397 people (114 cases, age 58.3±8.5, BMI 30.9±5.3; 284 controls, age 59.2±10.2, BMI 24.8±3.6). Training datasets were created by selecting 60% of the full dataset at random. Test datasets were created with the remaining 40% of the full dataset. Altogether, 20 random samples were selected creating 20 random training and test set combinations. For each of the training datasets, a generalised linear model was fitted with IR as the outcome and predictors including unadjusted DNA methylation levels and covariates (age, sex, BMI and smoker status) using the R glm function. The sensitivity and specificity were assessed using receiver operative curve (ROC), implemented using the pROC package in R [66]. Given the strong relationship between IR and BMI, we tested the performance of the methylation classifier compared to the performance of BMI alone. Additionally, we tested the performance of visceral fat alone. We tested whether there was a statistically significant difference between the two models using the ROC test function (pROC package). The test dataset was then loaded into the derived model with outcomes predicted using the R predict function and an average AUC was determined.

### Validation and replication

We pursed validation and replication of VF-DMPs and their relevance to metabolic health in three independent cohort samples (Additional file 2: Table S9).

We first sought to validate the 1181 VF-DMPs using AGR as a surrogate central adiposity phenotype. We fit linear models with AGR as predictor and DNA methylation levels at VF-DMPs as the response variable in the data set from Agha et al. [8]. The analysis included 104 individuals from the New England Family Study, the LEAP cohort (mean BMI 30.9 ± 7.03, mean age 47 ± 1.7, 48% male, mixed ancestry), described in detail previously [67]. SAT was collected from the upper outer quadrant of the buttock. DNA extraction and profiling, along with determination of AGR through DXA scanning has been previously described in detail [8]. For the validation analysis, a linear regression model was fit with covariates including age, sex, smoking status, BMI and batch effects. The analysis was carried out with and without adjustment for cell composition effects. The process for cell composition adjustment has previously been described [8] and is based on the reference-free method [68] with latent variables representing mean methylation. The latent variable dimension (i.e. number of cell types) was estimated to be 23, which in a sample of 104 may lead to potential overfitting and attenuation of effects. The primary results do not include adjustment for cell composition, and in the cell adjusted reference-free analysis, we observed that 51% (592) of tested VF-DMPs showed a consistent direction of methylation association with AGR in the LEAP cohort participants, and 2% displayed nominally significant effects with no results significant after multiple testing adjustment.

Second, we pursued replication of selected phenotype associations observed with the 19 VF-DMPs in 9 genes, which in our data showed significant associations with a wide range of metabolically relevant phenotypes, including insulin resistance, triglycerides, HDL and amino acid metabolites. We sought replication in two further cohort samples, a T2D case-control study from Nilsson et al. [22] and a set of unrelated obese individuals from Allum et al. [20].

The Nilsson et al. [22] Scandinavian case-control study dataset included 28 T2D cases (BMI 27.4±3.6, mean age 74.5±4.2, 46% female) and 28 controls (BMI 27.0±3.6; mean age 74.3±4.3; 46% female) from the Danish Twin Registry, described previously [22]. DNA extraction, methylation profiling and data quality control has been described previously [22]. Briefly SAT samples were extracted, frozen and stored at −80 °C. DNA was later extracted using DNeasy Blood and Tissue kit (Qiagen) and profiled on the 450k array. Of the 19 VF-DMPs, 18 CpG sites in 9 genes were available for replication testing. Relationships between T2D cases and controls were determined using paired Wilcoxon statistics and the resulting *P*-value compared with a Bonferroni threshold *P* = 2.8 × 10^−3^.

The final replication sample from Allum et al. [20] included 199 severely obese individuals (BMI > 40; mean age 37.2±8.8; 60% female) undergoing bariatric surgery at the Quebec Heart and Lung institute, Quebec City, Canada, for whom visceral adipose tissue samples were collected. DNA extraction, methylation profiling and data quality control have been described previously [20]. Briefly participants fasted overnight and underwent anaesthesia, and VAT samples were obtained within 30 min of the surgery from the greater omentum. In addition to the VAT samples, lipid levels (total cholesterol, HDL cholesterol, triglycerides) were measured in blood plasma using enzymatic assays. Plasma low-density cholesterol levels were estimated with Friedewald formula. In the analysis, lipid levels not normally distributed were transformed to a log scale. Methylation was profiled using Methyl C Capture Sequencing (MCC-seq), a targeted bisulfite sequencing epigenome profiling approach targeting 79.6Mb including 210,883 CpG sites from the 450k array as previously described [69]. We assessed the association between DNA methylation levels of specific targeted CpGs detected using the MCC-seq approach and the lipid levels using a generalised linear model fitted to a binomial distribution weighted for sequence coverage adjusted for age, sex, batch effects and BMI. Further details on this modelling have been previously reported [20]. If there was not an exact match to the target VF-DMP 450k array CpG site, we considered all MCC-seq signals within a 250-bp window around the target CpG site, reporting the most associated signal. Of the 19 VF-DMPs, there was an exact match for 11 CpG sites and at least one signal in the 250-bp window for 7 others.

## Results

**Fig. 1:**
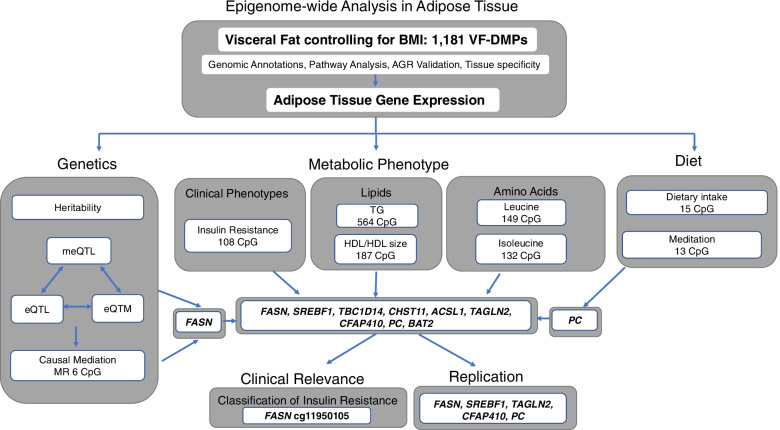
Study design. Epigenome-wide analyses of visceral fat (VF) were performed in 538 SAT samples, identifying 1181 VF-DMPs. The 1181 VF-DMPs were subject to downstream analyses assessing functional relevance using genomic annotations, tissue specificity, validation using a related phenotype android/gynoid ratio (AGR), and corresponding SAT gene expression changes related to VF. The VF-DMPs were then further analysed for association with genetic and lifestyle factors. Lastly, the signals were exploring using deep metabolic profiling across metabolic phenotypes, including lipid levels and metabolomic profiles, with replication. The resulting analyses were integrated to identify a set of replicated signals in genes with strong relevance to metabolic health

We characterised the SAT methylome signature of adiposity and metabolic disease risk, with integrative cross-omic follow-up and deep phenotype profiling (Fig. 1). The primary analysis aimed to investigate the relationship between SAT DNA methylation levels and VF. We sought to relate these findings to clinical outcomes including insulin resistance and T2D. The biological relevance of the findings was further explored through a deeper metabolic analysis of over 100 phenotypes including levels of lipids and amino acids. A detailed analysis plan is presented in Additional file 1: Fig. S1.

### Differential methylome signature associated with VF

**Fig. 2:**
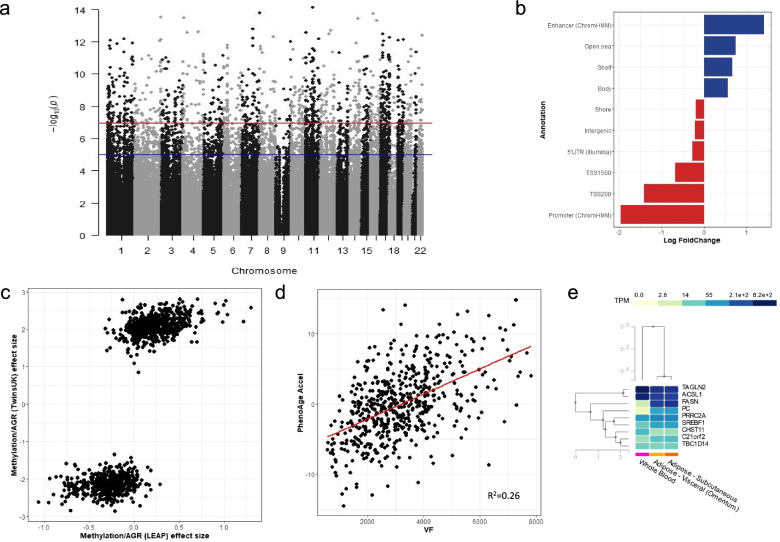
Subcutaneous adipose tissue differential methylation signature of VF. **a** A Manhattan Plot of the associations between VF and DNA methylation, taking into account BMI resulting from epigenome-wide association analysis (*N* = 538). The red line shows the multiple testing threshold (*P* = 1.14 × 10^−7^), and the blue line shows a relaxed significant threshold (*P* = 1 × 10^−5^). **b** Enrichment and depletion of VF-DMPs resulting from epigenome-wide association analysis (*N* = 538) across genomic annotations. Log Fold changes show the proportion of VF-DMPs annotated to particular genomic annotations, compared to all CpGs tested in each annotation. The plots show only annotation categories with significant enrichments and depletions. **c** Comparison between the discovery cohort (TwinsUK) (*N* = 538) and validation cohort (LEAP; *N* = 104) showing effect size for the associations resulting from the regression analysis between AGR and methylation at VF-DMPs without adjustment for cell composition in LEAP. **d** Significant positive association between PhenoAge acceleration and VF accumulation with a line of best fit shown in red along with its *R*^2^ value (*N* = 538). **e** GTEx gene expression levels in whole blood, visceral fat and subcutaneous fat for the 9 genes identified in the study following the omic integration showing shared expression levels in the two types of adipose tissue (SAT and VAT), but differences in expression levels between adipose tissue and whole blood. TPM is transcripts per kilobase million, and the median expression levels are shown in Additional file 2: Table S10

The association between VF, assessed using dual-energy X-ray absorptiometry (DXA) whole-body scans, and DNA methylation was explored in the primary dataset of 538 SAT samples from TwinsUK (Fig. 1, Table 1), using linear regression models correcting a number of covariates (see ‘Methods’). An epigenome-wide association analysis identified 1181 CpG sites (VF-DMPs) annotated to 788 genes that were statistically differentially methylated with VF, controlling for BMI, cell composition and further biological and technical covariates (see ‘Methods’), at a Bonferroni multiple testing threshold (*P* = 1.14 × 10^−7^; Additional file 2: Table S1; Fig. 2a). Of the 1181 VF-DMPs, 660 (56%) were hypermethylated with increasing VF, where VF-DMP effect sizes were estimated by repeating the association analysis without methylation normalisation. The effect sizes ranged in absolute value from less than 1% to up to 5% change in the unadjusted level of DNA methylation per kg of visceral fat, with a median of 1.6% methylation change per kg of visceral fat. The largest effect size was observed at cg23654401 (*VOPP1*), where methylation values were on average 5% lower per kg increase of visceral fat. The lowest *P*-value signal was obtained in *MAML3* (cg16218705; *P* = 2.4 × 10^−19^), which was previously reported to be differentially methylated with BMI in blood [7]. For reference, results without adjustment for BMI and association with BMI only are both shown in Additional file 2: Table S1.

The genomic distribution of the SAT VF-DMPs adjusted for BMI was explored across genomic annotations, including location with respect to gene body and CpG density (Fig. 2b). Consistent with previous observations that CpG islands (CGI) are less dynamic in response to exposures than surrounding regions [70], we observed a significant depletion of VF-DMPs in CGI (4% relative to 30% of probes, *P* = 9 × 10^−111^) and enrichment in CGI shores and shelves. The largest enrichment of VF-DMPs was obtained in enhancers, as predicted by ChromHMM (26% relative to 10% of probes tested, *P* = 3 × 10^−55^), showing clear links between accumulation of VF and changes in the regulatory genomic signature of SAT. This is consistent with previous investigations into methylation differences in regulatory regions for cardio metabolic traits [20].

Enrichment analyses explored biological pathways targeting the 1181 VF-DMPs and the 788 genes to which VF-DMPs were annotated. Using Ingenuity Pathway Analysis, we observed significant evidence for enrichment for 47 molecular pathways, which included many signalling pathways affecting metabolic health and cancer (Additional file 2: Table S2), and where the top pathway was Insulin Signalling (*P* = 4 × 10^−6^). KEGG pathway enrichment using GOmeth [43] for the 1181 VF-DMPs similarly identified significant enrichment for signals in genes involved in insulin signalling (*P* = 1.9 × 10^−3^), as well as glycolipid and fatty acid metabolism pathways.

We sought to validate the VF-DMPs by assessing methylation associations with other adipose phenotypes related to VFM, including VF/TFM and android:gynoid ratio (AGR), which has previously been linked to alterations in the SAT methylome in participants from the LEAP cohort [8]. We tested the association between AGR and DNA methylation levels, and between VF/TFM and DNA methylation levels, at the 1181 VF-DMPs after adjustment for BMI in the 538 participants from TwinsUK. All except one (cg00372886) of the 1181 VF-DMPs showed significant associations after multiple testing with VF/TFM with the same direction of effect as that observed for VF, and cg00372886 showed nominally significant associations. All the 1181 VF-DMPs showed significant associations after multiple testing adjustment with AGR (*P* = 4.2 × 10^−5^) with the same direction of effect as that observed for VF. A subset of 1172 VF-DMPs was then explored in the independent dataset of 104 younger male and female participants of mixed ethnicity from the LEAP cohort within the New England Family Study (mean BMI 30.9 ± 7.03, mean age 47 ± 1.7, 48% male), described in detail elsewhere [8]. We observed that 92% (1073 of 1172) of tested VF-DMPs showed a consistent direction of methylation association with AGR in the LEAP cohort participants (Fig. 2c), with 21% (220 of 1172) displaying nominally significant effects, with 9 sites significant after multiple testing correction (*P* = 4.2 × 10^−5^).

Two follow-up analyses explored the VF associated methylation signature, first, over larger genomic regions, and second, by minimising effects of genetic variation. First, we assessed the association between VF and DNA methylation levels over larger genomic regions, aiming to identify VF differentially methylated regions (VF-DMR) using DMRcate. Many VF-DMRs were identified after correction for multiple testing genome-wide, including cases where peak VF-DMRs overlapped peak VF-DMP signals such as in the *FASN* and *SREBF1* genes (Additional file 2: Table S11). Second, to minimise differential methylation effects attributed to genetic variation, we also carried out DNA methylation analyses in a sample subset of MZ twins with discordant VF levels, such that differences in twin VF levels exceeded 1sd of the VF distribution in our sample. MZ twins have very similar genetic variation profiles and are matched for age and sex effects. Altogether, only 7 MZ twin pairs showed discordant VF levels in our sample, and DNA methylation analyses within this subset alone identified both genome-wide and nominally significant MZ-specific signals (Additional file 3), including at sites identified from the main analyses, for example, for cg03498175 annotated to *ACSL1* (*P* = 7.8 × 10^−7^), cg11950105 annotated to *FASN* (*P =*1.8 × 10^−5^) and cg23875758 annotated to *SREBF1* (*P =* 3.4 × 10^−5^).

DNA methylation variation has been widely used to provide estimates of biological ageing measures across tissues, ages and species. We explored whether the accumulation of VF has an impact on biological ageing in SAT, by testing the association between three epigenetic ageing measures and VF. Epigenetic ageing measures included the estimated age acceleration rates for three predictors of age, lifespan and healthspan (Epigenetic Age Acceleration [40], GrimAgeAccel [38] and Levine’s PhenoAgeAccel [39]). The original Horvath Epigenetic Age accurately estimates chronological age and deviations from chronological age, while GrimAge and Levine’s PhenoAge have been proposed as different measures of lifespan and healthspan. In analyses not adjusting for BMI, all three epigenetic ageing measures in our SAT sample showed significant associations with VF, where the most significantly associated was PhenoAgeAccel (*P* = 2 × 10^−28^; GrimAgeAccel *P* = 1 × 10^−7^, Epigenetic Age Acceleration *P* = 6 × 10^−3^). After adjustment for BMI, only PhenoAgeAccel remained significantly associated with VF (*P* = 2 × 10^−6^; Fig. 2d).

### VF-DMP tissue specificity

This study was undertaken in SAT and identification of VF-DMPs took into account SAT cell composition variation. We sought to assess whether the 1181 VF-DMPs showed methylation differences between the SAT and the visceral adipose tissue (VAT) methylome in independent published datasets. In our first assessment, only 127 out of 1181 VF-DMPs (10.8%) fell in genomic regions that showed over 10% methylation differences between the SAT and VAT methylomes in an independent dataset of three SAT and VAT samples [44]. Our second comparison focused on SAT and VAT methylomes of 15 obese individuals [45], and only 5 of the 1181 VF-DMPs showed significant differences between tissue types. The results suggest that the majority of VF-DMPs in our SAT sample have similar DNA methylation profiles across VAT and SAT. In line with these methylation results, gene expression profiles from the Genotype-Tissue Expression (GTEx) resource were also highly consistent between SAT and VAT at VF-DMP genes, but the majority showed differences across SAT and whole blood samples (Fig. 2e, Additional file 1: Fig. S5, Additional file 2: Table S10). These findings provide a strong argument that the SAT-DMPs identified are reflective of VAT.

Tissue specificity of VF-DMPs was also explored between SAT and whole blood, in both the secondary TwinsUK dataset of 901 twins with blood methylation profiles and in a subset of 222 twins with both SAT and blood methylation profiles. Using the subset of 222 twins, we assessed the correlation between SAT and blood DNA methylation at the 1181 VF-DMPs. The majority of VF-DMPs (888, 75%) show very low correlation between blood and SAT methylation levels (−0.1<*r*<0.1), with only 2 sites reaching a correlation greater than 0.5 (Additional file 1: Fig. S2). We next assessed the association between whole blood DNA methylation and VF using the 901 whole blood samples. Of the 1181 VF-DMPs and after adjustment for BMI, there were no significant associations between blood methylation and VF (*π*0 = 1). Without adjustment for BMI, there was very weak evidence for blood methylation associations with BMI (*π*1 = 0.07), where 102 VF-DMPs showed nominally significant associations and no sites reached significance after multiple testing (*P* = 4 × 10^−5^). This suggests that the observed modest effects in blood are likely due to effects of BMI rather than specific impacts of VF accounting for BMI. These results provide evidence that the 1181 SAT VF-DMPs are predominantly not found in whole blood, and therefore may be adipose-specific or specific to the mesenchymal cell lineage.

### Subsets of VF-DMPs exhibit corresponding gene expression changes with VF

**Fig. 3:**
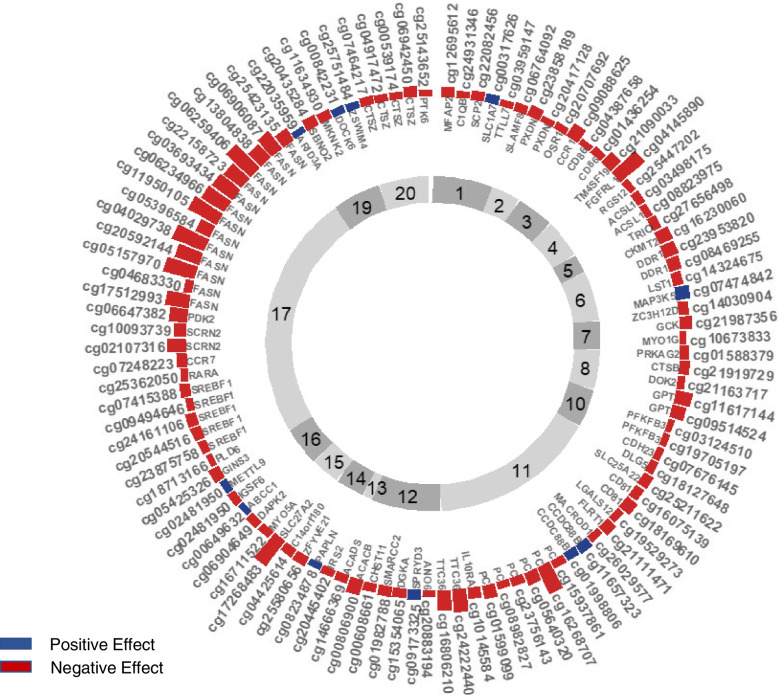
Significant subcutaneous adipose tissue DNA methylation and gene expression associations at VF-DMPs. The strength of association between DNA methylation and gene expression levels is shown from regression analysis (*N* = 538). There were 109 significantly associated CpG-Gene pairs, where CpGs are shown in the outer ring with the length of the bars showing −log10(*P*-value) which ranges from 4.3 to 22.6. Negatively associated pairs are in red, positively associated pairs are in blue. Gene names are shown inside the circle with the innermost ring showing the chromosome number

To characterize the functional signature of the SAT VF methylome signals, we explored SAT gene expression profiles at the 788 genes annotated to VF-DMP. We compared SAT DNA methylation and gene expression levels in the primary dataset of 538 twins, seeking to identify expression quantitative trait methylation signals (eQTMs). DNA methylation and expression levels were regressed at 848 CpG-gene pairs in *cis*, comprising 807 CpGs annotated to 717 genes after expression quality control assessments. Significant associations were observed at 109 CpG sites (13%) with 72 genes (10%) after multiple testing correction (Additional file 2: Table S3). The most significant correlation was obtained between SAT DNA methylation at cg04029738 (in *FASN*) and *FASN* expression levels (*P* = 2.5 × 10^−23^) where an increase in methylation leads to a decrease in expression. Methylation at a further 12 CpGs annotated to *FASN* also significantly associated with its expression levels (Fig. 3). Further significant methylation-expression associations were observed at multiple CpG sites, including in the *PC* (6 CpGs) and *SREBF1* (5 CpGs) genes.

We next investigated the association between SAT gene expression and VF at the 717 genes in the gene expression dataset of 720 TwinsUK participants. Pairwise gene expression and VF associations identified 227 genes (32%) to be significantly differentially expressed with VF after Bonferroni correction, with 415 genes (58%) at nominal significance. Next, conditional analyses explored whether methylation or expression was the likely driver of the association with VF. In the subset of 538 participants for which expression and methylation data were both available, 132 genes were significantly differentially expressed with VF after Bonferroni correction, leading to 174 CpG-gene pairs. The majority of these associations (66%) were no longer significant when methylation was included as covariate in the VF-expression linear model (Additional file 1: Fig. S3). In contrast, when the reverse analysis was undertaken at the 1181 VF-DMPs, only 12% of VF-DMPs were no longer significant if gene expression was included as a covariate in the VF-methylation linear model. The observation that the majority of VF-expression associations attenuate after conditioning on methylation suggests that methylation may likely be the driver of the association with VF at the majority (66%) of genomic regions that display both DNA methylation and gene expression associations with VF.

### Integrative genetic analyses highlight FASN

Obesity and metabolic health traits exhibit a clear genetic component, and multiple studies have now shown that a proportion of the human methylome is under strong influence of genetic variation. We assessed if the differential methylation signature of VF may be influenced by genetic variants, or DNA methylation quantitative trait loci (meQTLs). Due to the study participants being twins, we first applied twin-based heritability to explore evidence for genetic effects underlying DNA methylation variation in the primary dataset of 538 TwinsUK participants. Heritability results showed that 33% of the SAT VF-DMPs had evidence for substantial influence of additive genetic effects (A > 0.4). We then explored the association between genetic variation in *cis* and DNA methylation levels at the 1181 VF-DMPs in SAT, identifying 203 VF-DMP CpG sites (17%) with at least one genome-wide significant cis meQTL SNP (*P* = 1 × 10^−5^; Additional file 2: Table S4) in the primary dataset of 538 TwinsUK participants. We also carried out expression quantitative trait locus (eQTL) analysis in *cis* at the 717 genes in the larger dataset of 720 twins with SAT expression profiles. *Cis* eQTLs were identified at 125 genes (17%) with at least one genome-wide significant *cis* eQTL SNP (*P* = 1 × 10^−5^). Altogether, meQTLs were also eQTLs at a little more than a quarter (54, 27%) of the 203 VF-DMPs under genetic influence (Additional file 2: Table S4). For example, *FASN* had 211 eQTL SNPs that were also meQTL SNPs for *FASN* VF-DMP cg06906087.

We then assessed whether meQTL SNPs for VF-DMPs may also influence VF, integrating eQTL and eQTM findings. Our SAT meQTLs did not overlap with previously identified GWAS signals for VF or waist hip ratio adjusted for BMI [53]; therefore, we assessed the association between the 203 most associated meQTL SNPs and VF in the 538 individuals in this study. No SNP-VF associations were significant after multiple testing. Nominally significant SNP-VF associations were obtained with 8 meQTL SNPs, which were then used as instrumental variables in an exploratory Mendelian randomisation analysis to investigate the putative direction of association between VF and methylation at the corresponding VF-DMPs. At 6 VF-DMPs (in *FASN*, *TNFSF14*, *LPCAT1*, *FOXO1*, *CARS2*), we observed nominally significant evidence for putative causal effects of DNA methylation on VF (Additional file 2: Table S4). One of the 6 signals was cg06906087 in the *FASN* gene, which has previously been associated with insulin resistance [71]. Both *FASN* DNA methylation and gene expression (*P* = 3.2 × 10^−7^) were associated with VF in our study. In *FASN*, the instrumental variable SNP (rs34673303) exhibited both meQTL and eQTL effects, and there was a corresponding significant eQTM methylation-expression association. Further examples included cg04828493 at the *CARS2* gene, where the instrumental variable SNP was also both a meQTL and eQTL, as well as signals in other genes relevant to metabolic health including cg01684175 in *FOXO1* (involved in glucose regulation and adipogenesis) [72] and cg22185977 in *LPCAT1* (enzyme in the lipid-remodelling pathway) [73].

### Methylation mediates diet effects in VF

DNA methylation levels can change in response to environmental stimuli and diet is a major risk factor for metabolic health. To explore impacts of diet on VF-DMPs, we assessed DNA methylation associations at the 1181 VF-DMPs with 17 diet variables previously associated with VF accumulation [26] in the subset of 397 TwinsUK participants with diet data, which included specific nutrient intakes and indices of overall diet quality (see ‘Methods’). After multiple testing correction, 13 VF-DMPs (diet/VF-DMPs) exhibited differential methylation with fibre and magnesium intakes, and with three diet quality indices (DASH, DQI-I and Nordic diet score) (Additional file 2: Table S5). At the 13 DMPs, the direction of diet-DMP effect was consistent with the VF-DMP effect, such that a poorer diet score always corresponded to increasing VF (Additional file 1: Fig. S4). The diet/VF-DMPs annotated to 12 genes, with fibre, DASH and DQI-I all showing differential methylation in *PIK3R1* which is associated with insulin resistance [74]. The remaining 11 genes also included metabolically relevant genes such as *PC (*involved in insulin secretion [75]), *SLC2A2* (involved in glucose transport, linked to T2D [76]), *DGAT2* (involved in TG synthesis [77]) and others.

**Fig. 4:**
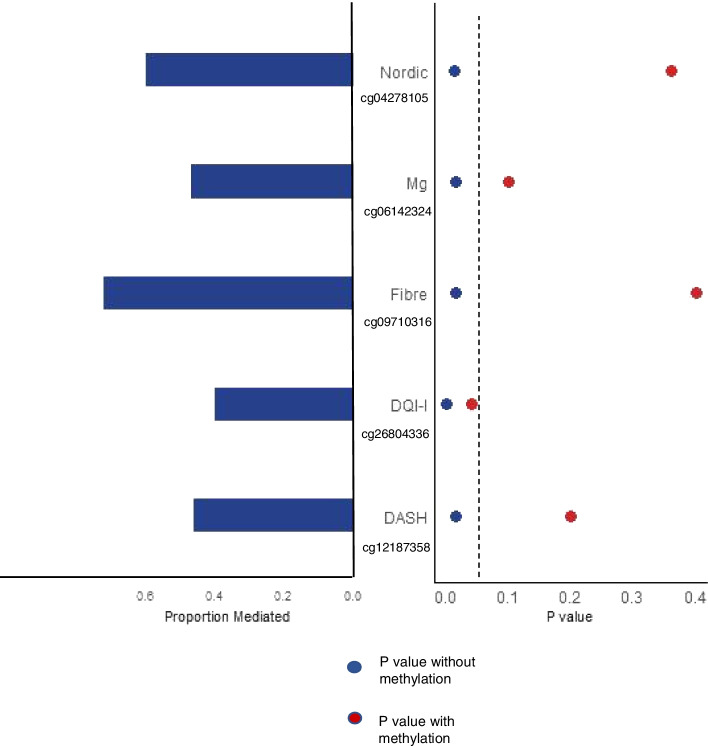
VF-DMPs link to diet. Impact of DNA methylation on the association between diet and visceral fat (*N* = 397). Left-hand side plots show the proportion of the effect mediated by methylation for each diet variable from the mediation analysis, and right-hand side shows the change in *P*-value when methylation is included in the association model from regression analysis. A vertical dotted line shows *P* = 0.05 (nominal significance). Results are shown for diet variables with significant associations with VF-DMPs

To assess whether DNA methylation may mediate the impact of diet on VF, we carried out mediation analyses focusing on diet variables that were significantly associated with both VF and the 13 diet/VF-DMPs. As expected, fibre and magnesium intake, and the three diet quality indices (DASH, DQI-I and Nordic diet score) all showed nominally significant associations with VF in our data (Additional file 2: Table S5) with DQI-I showing significance after multiple testing adjustment. We then tested whether the diet-VF associations were attenuated after inclusion of DNA methylation level as a covariate in the model. Following inclusion of DNA methylation as a covariate, all diet-VF associations were attenuated (Fig. 4a, Additional file 2: Table S5). Associations between VF and fibre, DASH and Nordic diet scores were all no longer nominally significant after accounting for DNA methylation levels at diet-CpGs. The association between magnesium and VF remained nominally significant after including DNA methylation levels at diet/VF-DMP cg19689330 (*FARS2*), with attenuation (original effect size = −1.7, standard error = 0.54, *P* = 1 × 10^−3^ *vs* methylation conditional effect size −1.2, standard error = 0.53, *P* = 0.03), but not after including methylation at cg06142324 (*HEPACAM*; methylation conditional *P* = 0.1). The association between DQI-I and VF remained nominally significant after including DNA methylation levels at diet/VF-DMP cg26804336 (*PIK3R1*) and cg20793665 (intergenic) in the model, although the strength of association was attenuated (original effect size = −14.6, standard error = 4.2, *P* = 6 × 10^−4^ *vs* methylation conditional effect size = −8.7 (cg26804336) and −11.1 (cg20793665), standard error = 4 (cg26804336 and cg20793665), *P* = 0.04 (cg26804336) and 0.008 (cg20793665)). A formal mediation analysis was then carried out with DNA methylation level at the most significantly associated CpG for each diet variable as the mediator between diet and VF, using R mediate [65]. We observed a full mediation effect of DNA methylation for VF associations with fibre, magnesium, DASH and Nordic diet scores, where only the average causal mediation effect (ACME) was significant (Fig. 4a, Additional file 2: Table S6). In total, methylation mediated 72% (cg09710316 in *SLC2A2*, *P* < 0.001) of the effect of fibre on VF, 47% (cg06142324 in *HEPACAM*, *P* < 0.001) of the effect of magnesium on VF, 46% (cg12187358 in *LAYN*, *P* = 0.004) of the effect of the DASH diet score and 60% (cg04278105 in *INF2*, *P* < 0.001) of the effect of the Nordic Score on VF. Only partial mediation was observed for the DQI-I diet score, where both ACME and the average direct effect (ADE) were significant (cg26804336 in *PIK3R1*, *P* < 0.001).

### Deep functional metabolic phenotype assessment of VF-DMPs

To explore potential chemical sequelae of the adiposity-related alterations to the SAT methylome, we assessed the association of SAT DNA methylation levels at the VF-DMPs with a large panel of blood lipid levels, serum metabolomic profiles and clinically relevant phenotypes such as insulin resistance (IR) and type 2 diabetes (T2D) in subsets of the 538 participants with relevant complete data.

We first linked the VF SAT methylation signatures to cardiometabolic disease risk, by relating the 1181 VF-DMPs to circulating serum lipid levels. Methylation associations with levels of triglycerides (TG), total cholesterol, high-density lipoprotein (HDL) and low-density lipoprotein (LDL) were assessed in 528 twins, controlling for BMI. Nearly half of the VF-DMPs (564) were significantly associated with TG allowing for multiple testing adjustment with a consistent positive direction of effect as for VF-DMPs, and nearly all (1125) were nominally significant. Similarly, 10% of VF-DMPs (109) were significantly associated with HDL allowing for multiple testing adjustment, with consistent opposite direction of effects as for VF-DMPs, and the majority (984) were nominally significant. *FASN* harboured the most significant signals for TG and HDL associations, and *SREBF1* also included top associated signals for TG.

**Fig. 5:**
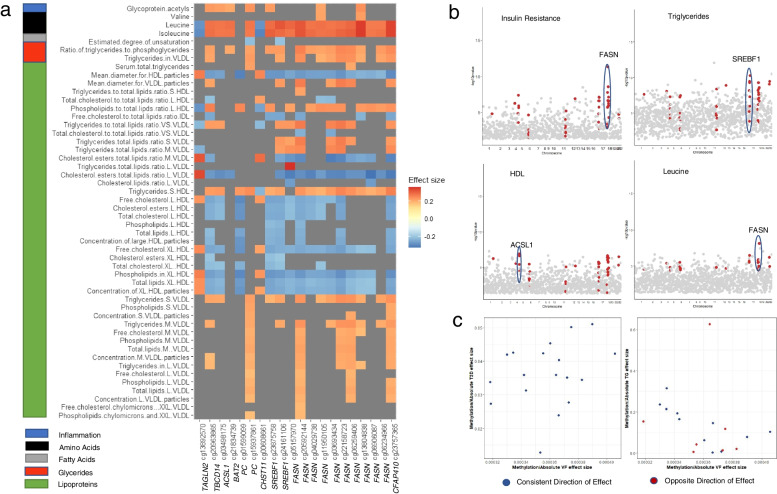
Deep functional metabolic phenotype analysis results. **a** Heatmap showing significant metabolite associations and their effect size from the regression analysis for the 19 VF-DMP CpGs in 9 genes identified in the multi-omic integration (*N* = 347). Only significant associations are shown, with grey areas reflecting correlations which did not meet the multiple testing significance threshold. Effect sizes for significant associations range from −0.33 to 0.36. **b** Nominally significant association results from regression analysis for IR (*N* = 397, 114 cases/284 controls), TG (*N* = 528), HDL (*N* = 528) and Leucine (*N* = 347) with the DNA methylation levels in the 9 genes identified in the multi-omic integration shown in red, in each case the gene containing the most significant result has been highlighted. **c** Replication results for subsets of the 19 VF-DMP CpGs tested by regression with T2D status (*N* = 56) in the Nilsson et al. [22] dataset (left), and with TG (*N* = 199) in the Allum et al. [20] dataset (right)

To further investigate the blood metabolic footprint of the VF-DMPs, we related DNA methylation levels at VF-DMPs to 239 fasting plasma and serum metabolites analysed using a nuclear magnetic resonance (NMR) metabolomics platform (Nightingale Health Ltd [28]) in a subset of 347 (of the 538) twins. The platform assays lipids and lipoprotein subclass profiles, fatty acids, amino acids, ketone bodies and glycolysis-related metabolites. Methylation-metabolite associations taking into account BMI identified significant associations in 71 (42%) lipoproteins and 2 (66%) lipoprotein sizes, 2 (22%) cholesterol metabolites, 3 (22%) glycerides/phospholipids, 1 (33%) apoliprotein, 5 (31%) fatty acid, 1 (20%) glycolysis-related metabolite and 6 (67%) amino acids (Additional file 2: Table S7). The majority of significant VF-DMPs metabolite associations were observed with leucine, isoleucine and HDL-related metabolites (Fig. 5a). As expected, most VF-DMPs exhibited negative associations with HDL, and positive associations with VLDL-related metabolites, in line with previously reported negative correlations between HDL and VF [78]. Similarly, the majority of VF-DMPs showed positive associations with isoleucine and leucine, in line with previous reports of increased levels of these amino acids with increased risk of T2D, including incident T2D [79].

**Fig. 6:**
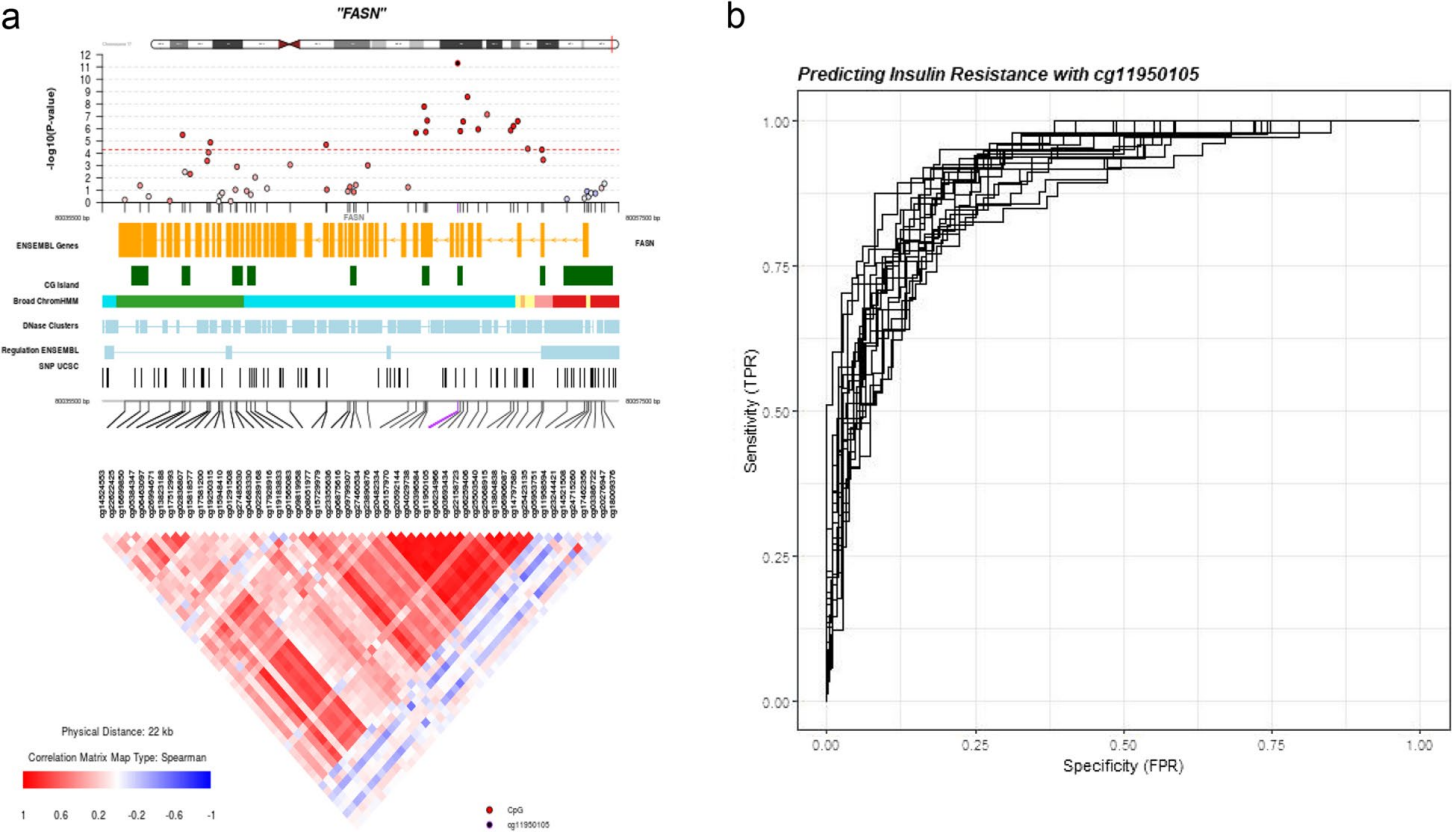
DNA methylation levels in FASN have predictive value for insulin resistance. **a** Epigenetic association from regression between VF and *FASN* DNA methylation (*N* = 538) displayed in a coMET plot [80], including VF-methylation association profiles (top panel) along with functional annotation of the region (middle panel), and pattern of co-methylation at the 53 CpG sites in the 450k array annotated to FASN (bottom panel). Broad ChromHMM regions are displayed using UCSC genome browser colour schemes. **b** ROC curves for insulin resistance based on unadjusted (not normalised) DNA methylation levels at cg11950105 (*FASN*) and age, smoking, BMI, SAT cell composition and technical covariates (*n* = 397, 114 cases/284 controls)

Finally, we assessed if the 1181 VF-DMPs methylation profiles capture variation in clinically relevant phenotypes, such as IR and T2D. Methylation associations between VF-DMPs and IR, controlling for BMI, were tested in a subset of 397 (114 IR cases / 284 IR controls) SAT samples. Altogether, 108 of the 1181 VF-DMPs (9%) showed a strong association with IR (IR/VF-DMPs) after multiple testing correction (*P* = 4.2 × 10^−5^), and the majority (822, 70%) showed nominal associations with a consistent direction of effect. The most significant IR-DMP was cg11950105 annotated to *FASN* (*P* = 5 × 10^−12^), with a further 14 sites in *FASN* also significantly associated with IR (Fig. 6a). In contrast to IR, our T2D sample was very small (15 T2D cases / 378 T2D controls) because the majority of the 538 female subjects with SAT biopsies were free from major disease. Correspondingly, we did not observe significant associations after multiple testing adjustment between VF-DMPs and T2D although at the majority of sites (840, 71%) the T2D-methylation effect matched the direction of the VF-methylation effect. Altogether, 4% of VF-DMPs (48 signals) were nominally associated with T2D with a consistent direction of association as that observed for VF-DMPs.

Integrating the deep metabolic phenotype methylation association results, we assessed overlaps between VF-DMPs that were also significantly associated with IR, circulating lipids and lipid and amino acid metabolomic signatures (Fig. 1, Fig. 5b). The results identified 19 VF-DMPs that annotate to 9 genes including *FASN*, *SREBF1*, *TBC1D14*, *CHST11*, *ACSL1*, *TAGLN2*, *CFAP410*, *PC* and *BAT2*, which leave a clear blood metabolic footprint and significantly associate with IR (Additional file 2: Table S8).

We pursued replication of the link between DNA methylation levels at the 19 VF-DMPs and metabolic health parameters in two independent cohort samples. The first replication set consisted of SAT samples from unrelated participants from the Danish Twin Registry, including 28 T2D cases (BMI 27.4±3.6, mean age 74.5±4.2, 46% female) and 28 controls (BMI 27.0±3.6; mean age 74.3±4.3; 46% female). Of the 19 VF-DMPs, 18 were tested and all showed a consistent direction of association with T2D (Fig. 5c) and were nominally significant (*P* < 0.05). Altogether, 5 signals (annotated to *FASN*, *SREBF1*, *TAGLN*, *PC*) replicated after multiple testing correction (*P* = 2.8 × 10^−3^). The second replication sample included visceral adipose tissue samples from 199 severely obese individuals of European ancestry (BMI > 40; mean age 37.2±8.8; 60% female) undergoing bariatric surgery, as previously described [20]. Replication analyses assessed the association between DNA methylation levels at the 19 VF-DMPs with circulating lipid levels of TG, HDL, LDL and total cholesterol. Of the 19 VF-DMPs, 18 were tested and 33–44% were nominally significant and matched direction of the discovery VF-DMP effect (Fig. 5c). Altogether, 4 VF-DMPs (annotated to *FASN and CFAP410*) replicated after multiple testing correction (*P* = 2.8 × 10^−3^). In summary, replication of the VF-DMP DNA methylation effects in metabolic health (T2D and circulating lipid levels) was obtained for VF-DMPs in 5 genes including *FASN*, *SREBF1*, *CFAP410*, *TAGLN* and *PC.*

### DNA methylation classifier of insulin resistance

Lastly, we investigated the extent to which DNA methylation levels could be used to classify insulin resistance. There was insufficient data to carry out the same analysis for T2D and no other clinical traits were considered in this study. Given the strong relationship between DNA methylation in *FASN* and IR, we focussed on this gene only. We assessed the performance of DNA methylation levels at *FASN* as a classifier of IR and therefore risk of metabolic disease in the primary dataset of TwinsUK participants. Given the strong relationship between IR and BMI, we assessed the performance of a classifier based on DNA methylation levels and BMI together, compared to the predictive power of BMI alone. Methylation at cg11950105 in *FASN* combined with BMI was a strong predictor of insulin resistance with an average AUC of 0.91 (Fig. 6b). The predictive value of this model was significantly greater than that for a prediction model including only BMI, which had an average AUC of 0.86 (*P*-values over 20 random training and test set combinations ranged from 0.001 to 0.03) or VF, which had an average AUC of 0.85.

## Discussion

In this study, we dissected the SAT methylation signature of visceral fat accumulation, a major risk factor for metabolic health. Our approach identified 1181 CpG sites in 788 genes to be differentially methylated in 538 discovery participants—one of the largest SAT samples to date [21], with validation and replication in 333 individuals from 3 independent adipose tissue samples. The vast majority of signals were not found in whole blood, annotating to genes strongly enriched for pathways relevant to metabolic health with the most significant result in the insulin signalling pathway. The VF-DMP signals were also validated for association with multiple adiposity measures in the discovery cohort and one replication cohort. We further found that visceral fat accumulation was significantly associated with accelerated biological ageing of SAT, which is one pathway through which VF may play a role in insulin resistance and metabolic disease. We integrated our visceral fat-associated SAT methylome results with genetic, blood methylation, adipose gene expression, blood metabolomic, diet and metabolic phenotype data, to identify and replicate signals in 5 genes (in *FASN*, *SREBF1*, *TAGLN2*, *PC*, *CFAP410*) that exhibit altered SAT function and have strong relevance to metabolic health. A subset of these methylation signals showed evidence for mediating effects of genetic variation and diet on VF, including signals in *FASN* and *PC*, respectively. Furthermore, *FASN* DNA methylation also exhibited putative causal effects on VF that were also strongly associated with insulin resistance, such that methylation in *FASN* was a better classifier of IR than BMI alone.

Overall, *FASN* had the strongest links to metabolic health where a number of CpG sites were associated with visceral fat, insulin resistance and metabolic signatures. *FASN* encodes an enzyme which catalyses the synthesis of palmitate, the most common saturated fatty acid found in animals. In *FASN*, genetic variants affected both DNA methylation and gene expression levels, which were also in turn both associated with VF, with evidence for putative causal methylation effects on increasing VF. The locations of differential methylation in *FASN* observed in our female-only sample are consistent with the SAT methylome associations with insulin resistance observed by Orozco et al. [10] in males, providing evidence for sex-shared effects of *FASN* on metabolic disease risk. Furthermore, SAT methylation levels in *FASN* were strongly predictive of insulin resistance status, significantly more so than BMI alone. Circulating FASN has been previously proposed as a biomarker for overnutrition-induced insulin resistance [71]. Whilst *FASN* effects in our study were only observed in SAT, and not blood, our findings are in line with previous reports of higher expression of *FASN* in adipose tissue linked to increased visceral fat and impaired insulin sensitivity [81]. Our hypothesis is that SAT DNA methylation levels of *FASN* likely reflect its DNA methylation levels in VAT and potentially other tissues such as liver, which we propose in turn impact its gene function in these metabolically relevant tissues, with downstream effects on metabolic health. The results also support a recently reported negative association [82] between adipose tissue *FASN* expression and serum levels of a novel family of endogenous lipids with anti-diabetic and anti-inflammatory effects, palmitic acid hydroxy stearic acids (PAHSAs) [83]. Our results confirm the key role of *FASN* in metabolic disease risk and provide insights into how specific genetic variants in this gene exhibit regulatory functional genomic effects that contribute to metabolic health.

Beyond *FASN*, our integrative methylation analysis with genetic and gene expression data also identified putative causal effects of methylation in *TNFSF14*, *LPCAT1*, *FOXO1*, *CARS2* on VF, and where *CARS2* DNA methylation and gene expression were also both under genetic control. These genes have all been shown to have important metabolic functions. *TNFSF14* encodes an inflammatory cytokine and enhanced levels of this cytokine are associated with T2D [84]. Mouse studies have also shown that deficiency in TNFSF14 improves liver glucose tolerance and reduces liver inflammation and non-alcohol fatty liver [85]. *LPCAT1* encodes an enzyme in the lipid-remodelling pathway [73] and knockdown *LPCAT1* mice have greater cytotoxicity due to excess polyunsaturated fatty acids [86]. *FOXO1* encodes a transcription factor in the insulin signalling pathway, regulating gluconeogenesis and glycolysis. Inhibition of FOXO1 in fat cells has been shown to mimic the impact of T2D and induce an insulin-resistant state [87]. Finally, whilst the role of *CARS2* in metabolic health regulation is less clear, it is a critical mitochondrial gene with a role in protein synthesis, charging tRNAs with amino acids. Whilst we observed a putative causal link between SAT methylation and visceral fat, there are many routes via which these associations may take place. One possibility is that genetic and environmental drivers of DNA methylation have similar effects across multiple tissues, including VAT, pancreas and others. Therefore, the methylation and gene expression changes observed in SAT could be surrogates for signals from other tissues, for example VAT, which may be the active mediators, including for potential dietary impacts. Overall, a third of the signals in our study displayed gene expression associations with VF, showing that not all of VF associated methylation signals have a corresponding association between gene function and VF. The remaining signals may capture signatures of previously accumulated ‘historic’ epigenomic variants, marking a progression in central adiposity changes in the body.

Diet is a key risk factor for metabolic health; therefore, we investigated how our visceral fat-associated methylation signals relate to dietary intakes previously associated with VF [26], revealing a subset of VF-DMPs also associated with fibre and magnesium nutrient intakes. We also identified VF-DMPs associated with diet quality scores (DASH, DQI-I, Nordic), where in line with expectation a poorer diet score always corresponded to increasing VF. The signals are in 12 genes including in metabolically relevant genes such as *PC*, *PIK3R1*, *SCL2A2* and *DGAT2*. *PC* encodes pyruvate carboxylase, an enzyme with an important role in gluconeogenesis. Reduced levels of PC are found in the islets of T2D patients and animal models have shown that blocking this enzyme reduced insulin secretion [75], highlighting a role of this gene in metabolic health. Additionally, *PC* has been suggested as a therapeutic target for insulin resistance [88]. *PIK3R1* encodes an enzyme with a direct role in insulin signalling and individuals with mutations in *PIK3R1* show severe insulin resistance [74]. For both *PIK3R1* and *SLC2A2* (which encodes a glucose transporter) genetic polymorphisms are associated with T2D [76]. Lastly *DGAT2*, is also an enzyme that catalyses the synthesis of triglycerides [77]. Methylation levels at a subset of diet/VF-DMPs in *SLC2A2* (cg09710316), *HEPACAM* (cg06142324), *INF2* (cg04278105) and LAYN (cg12187358) mediated the association between diet and VF, with up to 72% of dietary effect of VF mediated through methylation levels. One exception was the association between DQI-I and VF at cg26804336 in *PIK3R1*; however, this same CpG site showed a mediating effect in the relationship between the DASH diet score and VF and the relationship between fibre and VF. One of the diet/VF-DMPs in the pyruvate carboxylase gene (*PC*, cg01599099) also showed strong associations in subsequent analysis with multiple deep metabolic phenotypes. Given these associations, a formal mediation analysis was carried out and showed that methylation at cg01599099 (*PC*) mediated 35% of the association between the DASH diet score and VF (Additional file 2: Table S6).

To assess the functional and clinical phenotypes reflecting the adiposity-related alterations to the SAT methylome, we related VF-DMPs with a large panel of blood lipid levels, serum metabolomic profiles, insulin resistance (IR) and type 2 diabetes (T2D). The results identified marked strong associations between the 1181 VF-DMPs and IR. In addition to *FASN*, strong associations were seen for sites within *SREBF. SREBF1* has consistently been reported to exhibit differential methylation with T2D in blood [89]. However, *SREBF1* was not found to be associated with adiposity measures in other recent adipose methylation studies [10, 11]. The metabolomic profiling relationships further identified strong VF-DMP associations with HDL and with leucine. There is evidence that leucine supplementation may offer therapeutic possibilities in metabolic-related disorders [90]. Effects observed from dietary supplementation of leucine include improved lipid and glucose metabolism. Previous studies have found circulating levels of branched chain amino acids including isoleucine and leucine to be positively associated with measures of adiposity [91] and T2D [92]. Integrating the results from all the metabolic and clinical phenotypic analyses identified 19 VF-DMPs that annotate to 9 genes, of which 5 achieved replication (*FASN*, *SREBF1*, *PC*, *TAGLN2* and *CFAP410)*. Aside from *FASN*, *SREBF1* and *PC*, all of which have been previously linked to metabolic health, previous work has shown that *TAGLN2* levels are increased in obese adipose tissue [93].

There are several limitations to the current study. Ideally, this study would be undertaken in visceral adipose tissue (VAT). However, VAT samples are not typically available for healthy research participants due to the invasive nature of the biopsy required. The VF-DMP signals we obtained showed overall concordant DNA methylation levels across SAT and VAT samples, with consistent gene expression changes. Although our discovery sample is among the largest adipose tissue methylation datasets profiled so far [21], a sample of 538 is nevertheless relatively modest for epigenetic analyses aiming to identify moderate to small effects. The Mendelian randomisation analysis included in the paper was limited by the sample size and more robust conclusions would be drawn from larger sample sizes. Our sample consisted of female-only White British twins, and therefore, results may not translate to males and to other ethnicities, although a proportion of our findings were replicated in males and in mixed sex cohort samples. The SAT samples were also predominantly from individuals who were not affected by major disease [21], which may miss epigenetic changes relating to obesity-related disease. Utilising a Bonferroni adjustment for multiple testing is a conservative approach as it assumes that DNA methylation levels at CpG sites are independent of each other, while multiple studies have reported evidence for co-methylation [94]. Furthermore, although the key clinical phenotypes were obtained at time of adipose tissue biopsy in the discovery dataset, the metabolomic profiles were obtained within up to 5 years of time of biopsy sampling, where the time differences may limit the interpretation of these results. A natural extension to this work would be to carry out a longitudinal follow-up and to explore how baseline methylation levels and their longitudinal trajectory relate to metabolic disease incidence, to better disentangle cause from effect at the identified changes.

## Conclusions

In conclusion, the SAT methylome shows a distinct epigenetic signature associated with visceral fat after controlling for BMI. Integrating genetic, gene expression, metabolomics, diet and metabolic traits highlights five genes after replication, with strong relevance to metabolic disease mechanisms. Our findings may help to further understand the regulatory genomic pathways underlying metabolic disease risk.

## Supplementary Information

**Additional file 1: Supplementary Figures 1 – 5.**

**Additional file 2: Table S1.** Statistically significant adipose tissue CpGs in relation to visceral fat accumulation at Bonferroni 5% (VF-DMPs). **Table S2.** Pathway Analysis (IPA) results. **Table S3.** Statistically significant associations between methylation and gene expression at VF-DMPs. **Table S4.** meQTLs. **Table S5.** Diet VF-DMPs. **Table S6.** Diet-VF mediation by methylation. **Table S7.** Effect sizes for statistically significant associations with metabolites. **Table S8.** Metabolic Phenotype results and Replication. **Table S9.** Validation and Replication Cohort Characteristics. **Table S10.** GTEx median expression levels in SAT, VAT and Whole Blood. **Table S11.** Results from Differentially methylated region analysis.

**Additional file 3: Supplementary Note 1.** MZ Discordant Twins Analysis.

**Additional file 4.**

## Data Availability

TwinsUK methylation datasets analysed in the current study is available under ArrayExpress accession number E-MTAB-1866 (https://www.ebi.ac.uk/arrayexpress/experiments/E-MTAB-1866/) [31]. The expression dataset analysed in the current study is available under EGA accession number EGAS00001000805 (https://ega-archive.org/studies/EGAS00001000805) [48]. ‘Additional individual-level data are not permitted to be publicly shared or deposited due to the original consent given at the time of data collection, where access to these data is subject to governance oversight. All data access requests are overseen by the TwinsUK Resource Executive Committee (TREC). For information on access to these genotype and phenotype data and how to apply, see https://twinsuk.ac.uk/resources-for-researchers/access-our-data/.’ The code for carrying out the EWAS and for the mediation analysis has been added to Github (https://github.com/colette1nz/EWAS) [95].
