# Supplementary material for: Adipose methylome integrative-omic analyses reveal genetic and dietary metabolic health drivers and insulin resistance classifiers
Source: Genome Med. 2022 Jul 18;14:75. doi: 10.1186/s13073-022-01077-z (PMC9290282; doi:10.1186/s13073-022-01077-z)
Supplement: Supplementary file 1 — Additional file 1: Supplementary Figures 1 – 5. [file 13073_2022_1077_MOESM1_ESM.ppt]

## Slide 1
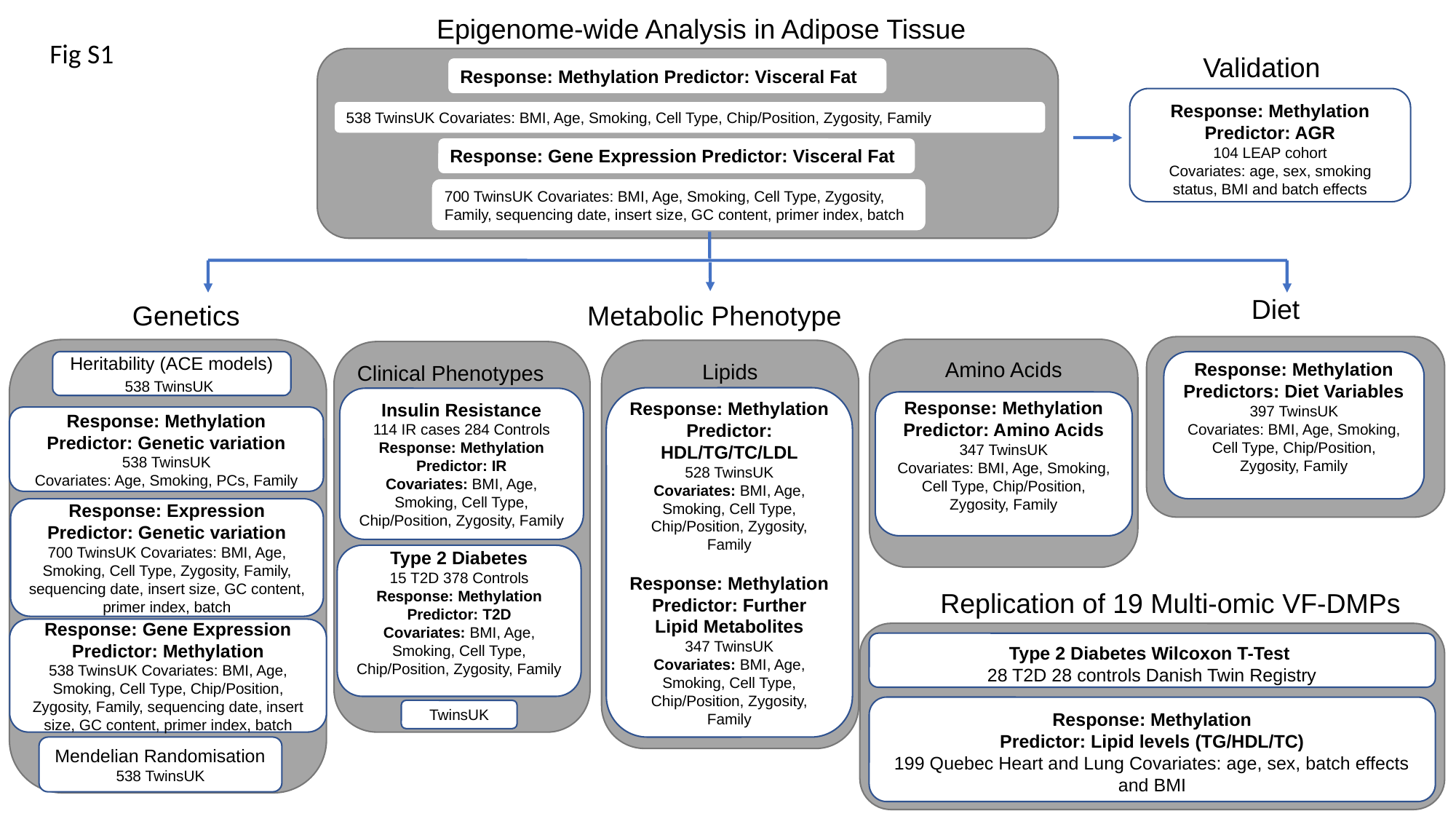

Epigenome-wide Analysis in Adipose Tissue
Fig S1
Validation
Response: Methylation Predictor: Visceral Fat
Response: Methylation Predictor: AGR
104 LEAP cohort
Covariates: age, sex, smoking status, BMI and batch effects
538 TwinsUK Covariates: BMI, Age, Smoking, Cell Type, Chip/Position, Zygosity, Family
Response: Gene Expression Predictor: Visceral Fat
700 TwinsUK Covariates: BMI, Age, Smoking, Cell Type, Zygosity, Family, sequencing date, insert size, GC content, primer index, batch
Diet
Genetics
Metabolic Phenotype
Amino Acids
Lipids
Clinical Phenotypes
Heritability (ACE models)
538 TwinsUK
Response: Methylation Predictors: Diet Variables
397 TwinsUK
Covariates: BMI, Age, Smoking, Cell Type, Chip/Position, Zygosity, Family
Response: Methylation
Predictor: HDL/TG/TC/LDL
528 TwinsUK
Covariates: BMI, Age, Smoking, Cell Type, Chip/Position, Zygosity, Family
Response: Methylation Predictor: Further Lipid Metabolites
347 TwinsUK
Covariates: BMI, Age, Smoking, Cell Type, Chip/Position, Zygosity, Family
Insulin Resistance
114 IR cases 284 Controls
Response: Methylation Predictor: IR
Covariates: BMI, Age, Smoking, Cell Type, Chip/Position, Zygosity, Family
Response: Methylation Predictor: Amino Acids
347 TwinsUK
Covariates: BMI, Age, Smoking, Cell Type, Chip/Position, Zygosity, Family
Response: Methylation Predictor: Genetic variation
538 TwinsUK
Covariates: Age, Smoking, PCs, Family
Response: Expression Predictor: Genetic variation
700 TwinsUK Covariates: BMI, Age, Smoking, Cell Type, Zygosity, Family, sequencing date, insert size, GC content, primer index, batch
Type 2 Diabetes
15 T2D 378 Controls
Response: Methylation Predictor: T2D
Covariates: BMI, Age, Smoking, Cell Type, Chip/Position, Zygosity, Family
Replication of 19 Multi-omic VF-DMPs
Response: Gene Expression Predictor: Methylation
538 TwinsUK Covariates: BMI, Age, Smoking, Cell Type, Chip/Position, Zygosity, Family, sequencing date, insert size, GC content, primer index, batch
Type 2 Diabetes Wilcoxon T-Test
28 T2D 28 controls Danish Twin Registry
Response: Methylation
Predictor: Lipid levels (TG/HDL/TC)
199 Quebec Heart and Lung Covariates: age, sex, batch effects and BMI
TwinsUK
Mendelian Randomisation
538 TwinsUK

## Slide 2
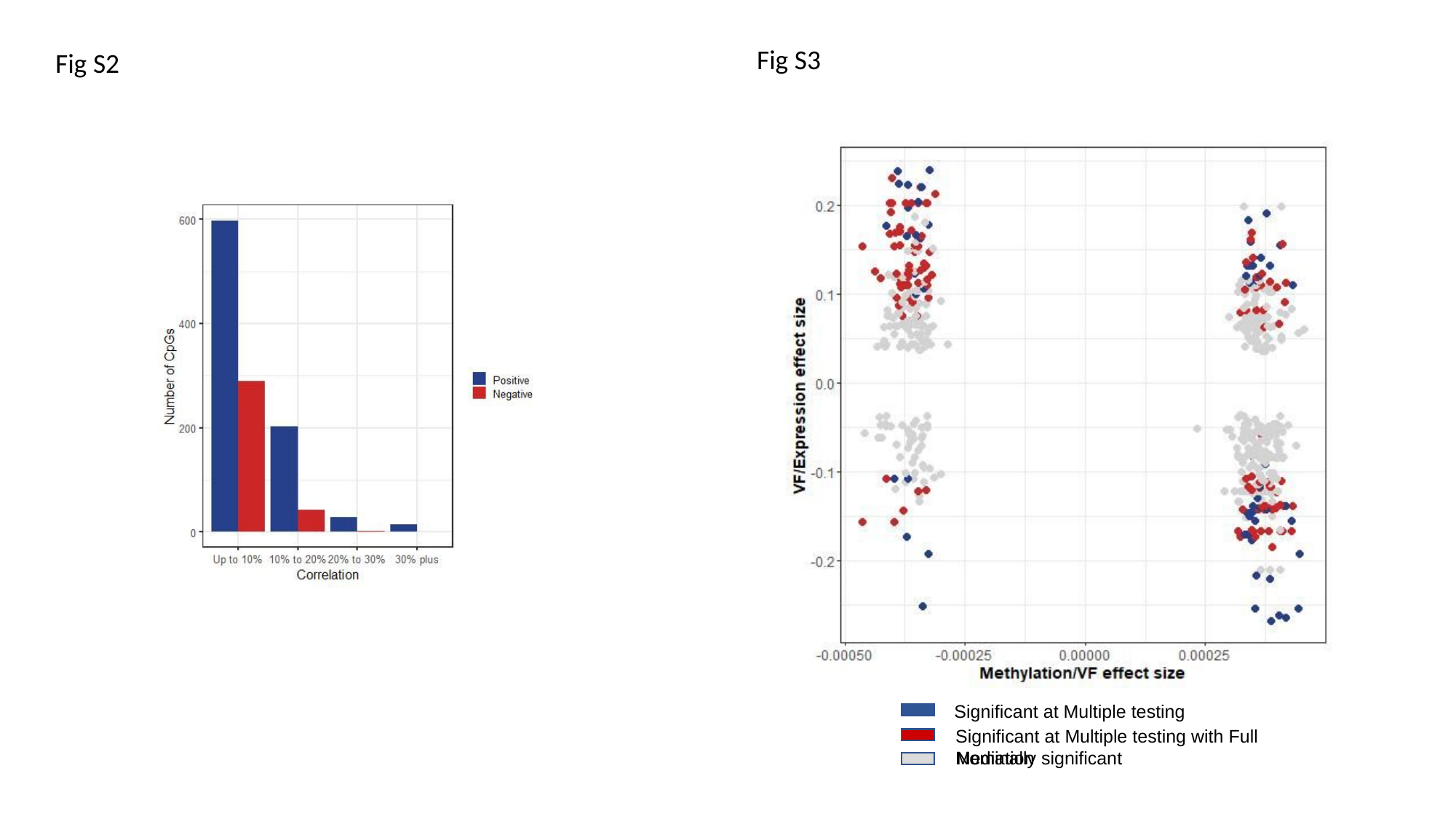

Fig S3
Fig S2
Significant at Multiple testing
Significant at Multiple testing with Full Mediation
Nominally significant

## Slide 3
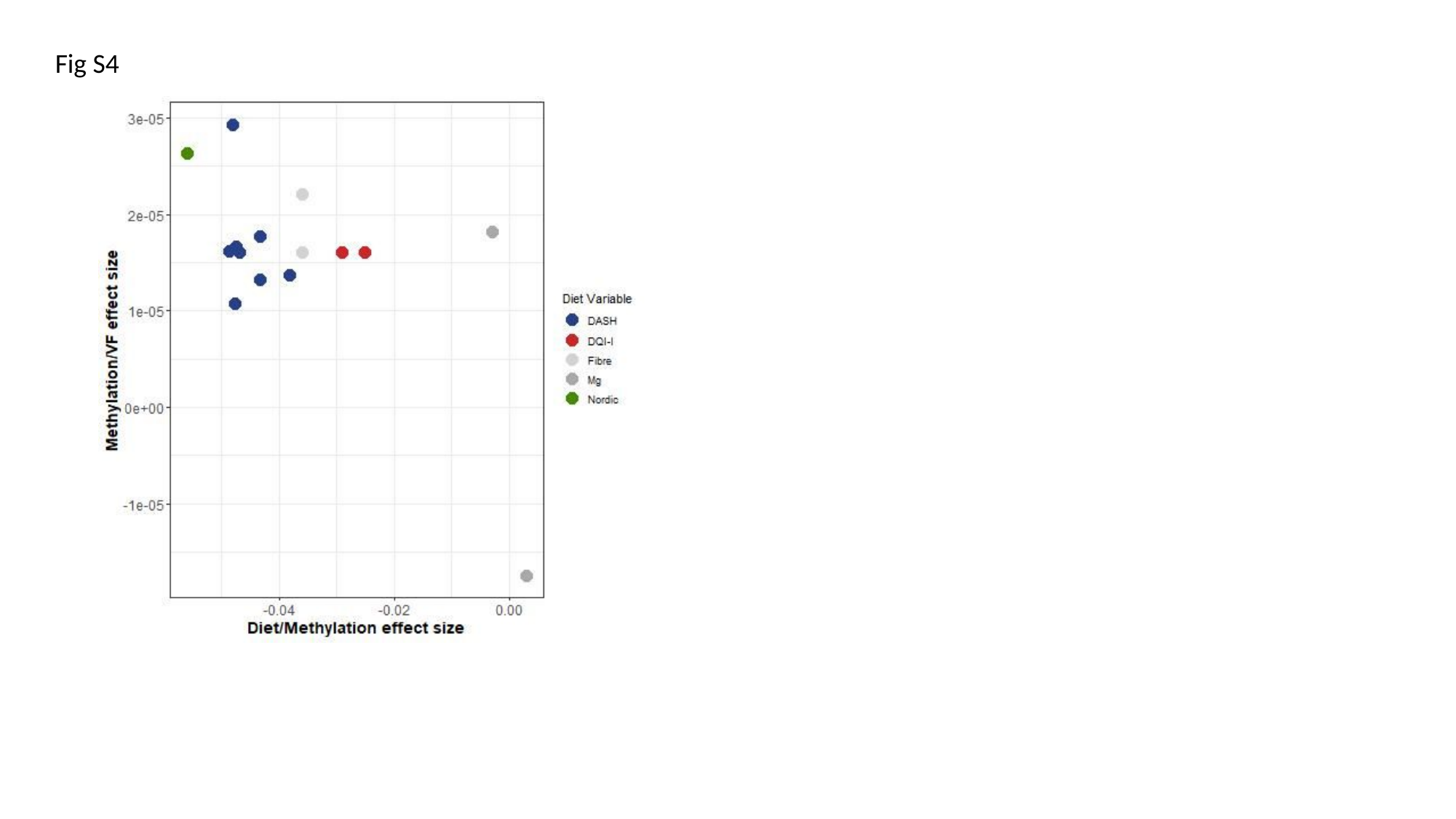

Fig S4

## Slide 4
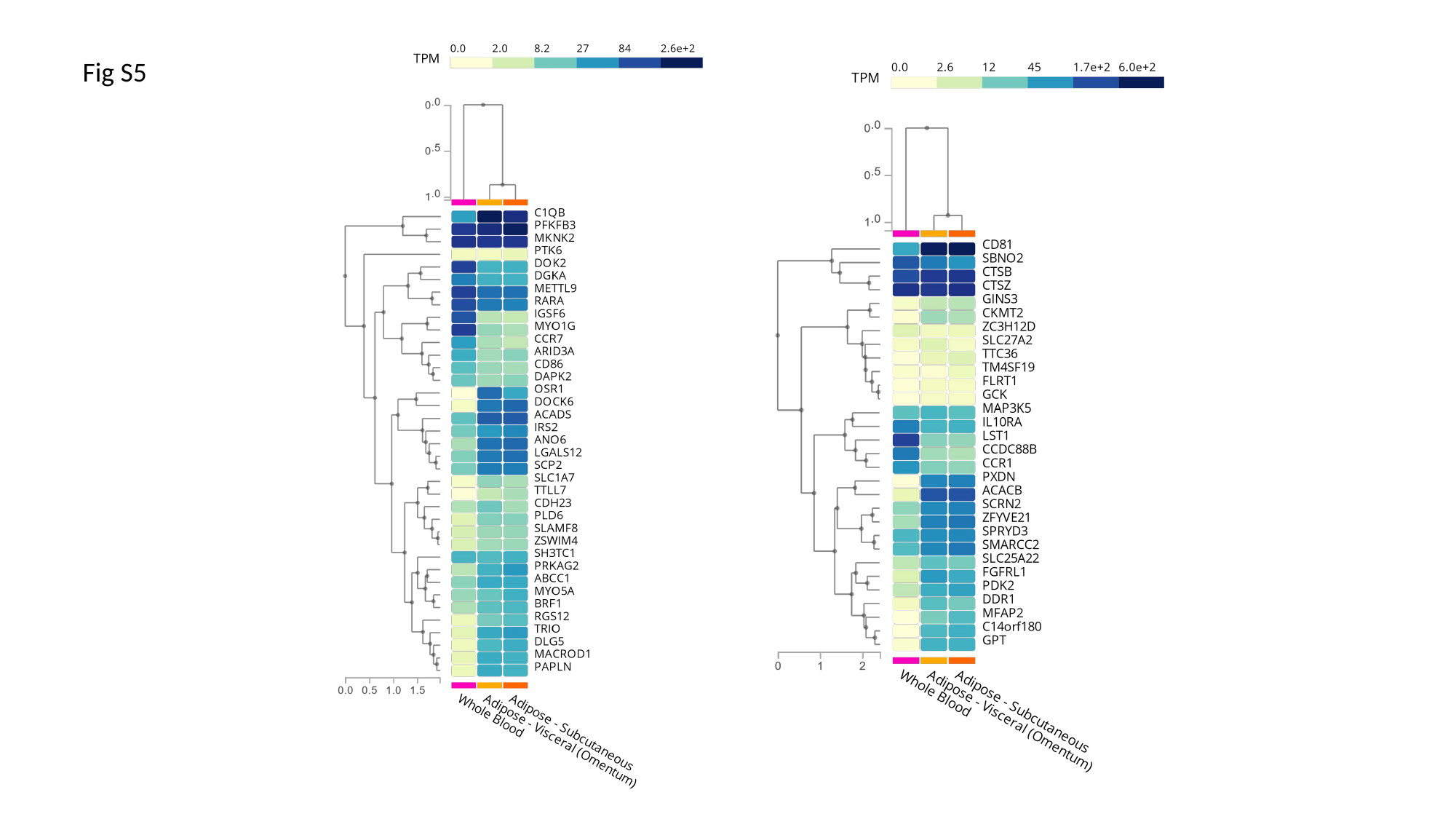

Fig S5
