# Supplementary material for: Adipose methylome integrative-omic analyses reveal genetic and dietary metabolic health drivers and insulin resistance classifiers
Source: Genome Med. 2022 Jul 18;14:75. doi: 10.1186/s13073-022-01077-z (PMC9290282; doi:10.1186/s13073-022-01077-z)
Supplement: Supplementary file 3 — Additional file 3: Supplementary Note 1. MZ Discordant Twins Analysis. [file 13073_2022_1077_MOESM3_ESM.pdf]

Supplementary Material 1: MZ discordant analysis

Twin pairs were analysed to establish the difference between their visceral fat levels. The standard deviation of visceral fat for all participants was determined to be 1382g. Twin pairs that differed in visceral fat levels by more than 1382 (1sd) were considered to be discordant. This identified 48 twin pairs that were discordant with whole blood methylation profiles and 7 twin pairs that were discordant with adipose tissue methylation profiles. Methylation profiles were normalised and a pair wise t-test was then carried out on the methylation levels between the high visceral fat twin and the low visceral fat twin to identify CpG sites that were differentially methylated.

Discordant twin pair results

Despite the small sample size, 10 CpG sites in 9 genes were differentially methylated at FDR <0.05 (Table 1), one of these cg03498175 (*ACSL1*) was identified in the main analysis. This gene also contains SNPs previously associated with Type 2 Diabetes in GWAS. The remaining genes fell into three categories, relating to mental health – that may potentially contribute towards adiposity development, relating to cell proliferation – potentially arising because of adiposity, and relating to cancer.

CpG	P value	Estimate	FDR	Gene	Category
**cg16361867**	3.92E-08	1.24	0.009	*GRIK4*	Mental Health
**cg16001422**	4.09E-08	-1.02	0.009	*PLEC1*	Cell Proliferation
**cg07491702**	2.16E-07	-1.00	0.0318	*CDAN1*	Mental Health
**cg10617782**	4.11E-07	-1.29	0.0323	*Chr7*	
**cg23654821**	4.44E-07	-1.35	0.0323	*CSRNP1*	Cancer
**cg14962774**	5.20E-07	-1.40	0.0323	*GDAP2;WDR3*	Cancer
**cg25590444**	6.30E-07	1.35	0.0323	*Chr1*	

**cg04576707**	6.70E-07	-1.08	0.0323	*MOBKL1A*	Cell proliferation/Type 2 diabetes GWAS
**cg03498175**	7.77E-07	0.93	0.0323	*ACSL1*	Type 2 diabetes GWAS
**cg05429895**	8.03E-07	-1.24	0.0323	*TLR4*	Mental Health

The results for the 19 VF-DMPs identified from the integrated omics analysis as shown in the table below.

**CpG**	**Gene**	**Pvalue**	**Estimate**
**cg06906087**	FASN	0.000101	1.117408
**cg20963865**	TBC1D14	0.000912	1.105669
**cg20592144**	FASN	0.00336	1.114668
**cg00608661**	CHST11	0.001608	-1.2207
**cg11950105**	FASN	1.76E-05	0.931996
**cg24161106**	SREBF1	0.000115	0.943561
**cg05157970**	FASN	0.015564	0.974881
**cg04029738**	FASN	0.000107	1.236273
**cg23875758**	SREBF1	3.43E-05	1.30008
**cg06259406**	FASN	0.000589	1.114668
**cg03498175**	ACSL1	7.77E-07	0.931996
**cg13892570**	TAGLN2	0.328359	-0.29274
**cg22158723**	FASN	0.003174	1.054444
**cg23757365**	C21orf2	0.000765	0.895501
**cg13804838**	FASN	0.000304	1.010532
**cg03693434**	FASN	0.028489	0.737401
**cg21834739**	BAT2	0.017966	0.970873
**cg01599099**	PC	0.052299	0.863997

**cg06234966**	FASN	0.035947	0.661604
