# Supplementary material for: Adipose methylome integrative-omic analyses reveal genetic and dietary metabolic health drivers and insulin resistance classifiers
Source: Genome Med. 2022 Jul 18;14:75. doi: 10.1186/s13073-022-01077-z (PMC9290282; doi:10.1186/s13073-022-01077-z)
Supplement: Supplementary file 4 — Additional file 4. [file 13073_2022_1077_MOESM4_ESM.pdf]

Supplementary Material – Code

Code for Epigenome-wide Association Analysis

```
#Load libraries

library(lme4)

library(lmerTest)

options(stringsAsFactors=FALSE)

#treat strings in a data frame as characters and not as factors by default


#load data

fulldata <-[Read in file with ID, Predictor, Covariates and Methylation all in columns]

methdata <-[Read in file with ID and Methylation in columns]


#normalise methylation data

normdataC <-function(x){normdat <-qqnorm(x,plot.it=FALSE)

resultnorm <-normdat$x}

normdata <-apply(methdata,2,normdataC)


#linear model function

runlinmod <- function(x){

  y <-normdata[,x]

  h<-lmer(y~[Predictor] +[Covariates],data=fulldata,na.action=na.omit)

  coeff <-summary(h)$coefficients

  result <-c(coeff[2,1],coeff[2,2],coeff[2,4],coeff[2,5])

  return(result)

}


modelres <-do.call(cbind,lapply(1:NCOL(normdata),runlinmod))

rownames(modelres) <-c("estimate","S.error","t value","pvalue")

colnames(modelres) <-colnames(normdata)
```

```
modelresout <-t(modelres)

write.csv (modelresout, file=[location])
```

Code for mediation analysis

```
diet_input<-[Read in file with diet data, visceral fat, covariates and methylation data]

M <-diet_input$cg26804336

Y <-as.numeric(diet_input$VF)

#model.0 <-lm(Y~X)

model.0<-lmer(Y~dqii_score + BMI+ as.factor(smokestatus1) + Age+ (1|KCLfam),data=diet_input)

coeff0 <-summary(model.0)$coefficients

#model.1 <-lm(M~X)

meth_model<-lmer(M~dqii_score + BMI+ as.factor(smokestatus1) + Age + (1|KCLfam),
data=diet_input)

coeff1 <-summary(meth_model)$coefficients

#model.Y <-lm(Y~X+M)

meth_diet_model<-lmer(Y~dqii_score + cg26804336+ BMI+ as.factor(smokestatus1) + Age+
(1|KCLfam),data=diet_input)

coeff2 <-summary(meth_diet_model)$coefficients

library(mediation)

results <-mediate(meth_model, meth_diet_model,sims =500,treat="dqii_score",
mediator="cg26804336", boot=FALSE)

summary(results)
```
